# Review of Intelligence for Additive and Subtractive Manufacturing: Current Status and Future Prospects

**DOI:** 10.3390/mi14030508

**Published:** 2023-02-22

**Authors:** M. Azizur Rahman, Tanveer Saleh, Muhammad Pervej Jahan, Conor McGarry, Akshay Chaudhari, Rui Huang, M. Tauhiduzzaman, Afzaal Ahmed, Abdullah Al Mahmud, Md. Shahnewaz Bhuiyan, Md Faysal Khan, Md. Shafiul Alam, Md Shihab Shakur

**Affiliations:** 1 Department of Mechanical and Production Engineering, Ahsanullah University of Science and Technology, Dhaka 1208, Bangladesh; 2 McMaster Manufacturing Research Institute (MMRI), Department of Mechanical Engineering, McMaster University, Hamilton, ON L8S4L7, Canada; 3 Autonomous Systems and Robotics Research Unit (ASRRU), Department of Mechatronics Engineering, International Islamic University Malaysia (IIUM), Kuala Lumpur 53100, Malaysia; 4 Department of Mechanical and Manufacturing Engineering, Miami University, Oxford, OH 45056, USA; 5 Department of Mechanical Engineering, National University of Singapore, Singapore 117575, Singapore; 6 Singapore Institute of Manufacturing Technology, 73 Nanyang Drive, Singapore 637662, Singapore; 7 National Research Council of Canada, 800 Collip Circle, London, ON N6G 4X8, Canada; 8 Department of Mechanical Engineering, Indian Institute of Technology Palakkad, Palakkad 678557, India; 9 School of Design, Swinburne University of Technology, Melbourne, VIC 3122, Australia; 10 Department of Mechanical Engineering, Auburn University, Auburn, AL 36849, USA; 11 Department of Industrial & Production Engineering, Bangladesh University of Engineering & Technology (BUET), Dhaka 1000, Bangladesh

**Keywords:** intelligent manufacturing, digital twin, feedback control, smart system, data analytics

## Abstract

Additive manufacturing (AM), an enabler of Industry 4.0, recently opened limitless possibilities in various sectors covering personal, industrial, medical, aviation and even extra-terrestrial applications. Although significant research thrust is prevalent on this topic, a detailed review covering the impact, status, and prospects of artificial intelligence (AI) in the manufacturing sector has been ignored in the literature. Therefore, this review provides comprehensive information on smart mechanisms and systems emphasizing additive, subtractive and/or hybrid manufacturing processes in a collaborative, predictive, decisive, and intelligent environment. Relevant electronic databases were searched, and 248 articles were selected for qualitative synthesis. Our review suggests that significant improvements are required in connectivity, data sensing, and collection to enhance both subtractive and additive technologies, though the pervasive use of AI by machines and software helps to automate processes. An intelligent system is highly recommended in both conventional and non-conventional subtractive manufacturing (SM) methods to monitor and inspect the workpiece conditions for defect detection and to control the machining strategies in response to instantaneous output. Similarly, AM product quality can be improved through the online monitoring of melt pool and defect formation using suitable sensing devices followed by process control using machine learning (ML) algorithms. Challenges in implementing intelligent additive and subtractive manufacturing systems are also discussed in the article. The challenges comprise difficulty in self-optimizing CNC systems considering real-time material property and tool condition, defect detections by in-situ AM process monitoring, issues of overfitting and underfitting data in ML models and expensive and complicated set-ups in hybrid manufacturing processes.

## 1. Introduction

**Figure 1:**
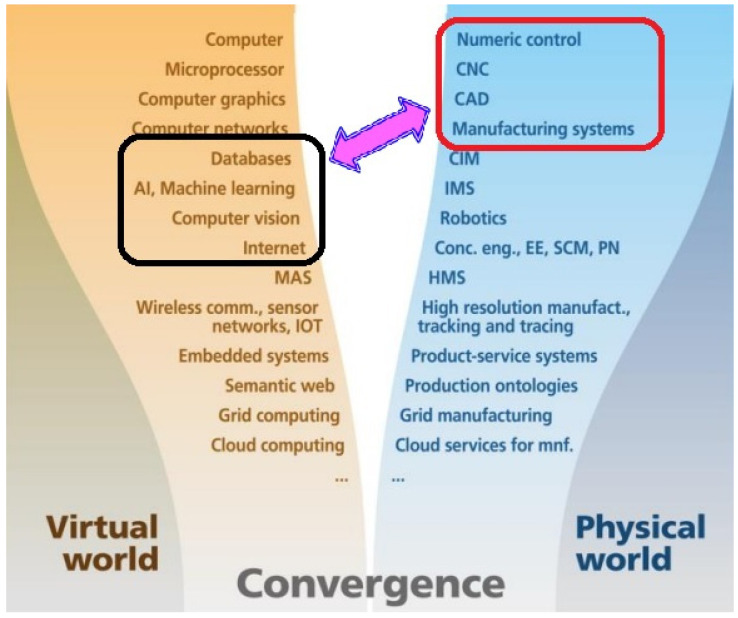
Interplay between computer science, information communication technology (ICT) and manufacturing (reprinted with permission from [2]. Copyright 2016 CIRP).

The manufacturing processes are becoming increasingly complex, dynamic and networked as industrialization progresses toward global connections [1]. Thus, significant progress toward the cyber physical systems (CPS) through the internet-of-things (IoT) with the integration of sensors, data analytics, automation and robotics is leading to the pervasive digitalization of the factory operations. Therefore, enterprises are adopting innovation in computing, information systems and communication technology to converge with manufacturing science and production technology, as observed in the parallel development of the virtual and physical world, as illustrated in Figure 1 [2]. Moreover, adopting cloud technologies enables delivering and receiving services via an intelligent system leading to cloud manufacturing (CMfg) [3]. In this regard, the blockchain technology can be an enabler in the manufacturing industry, specifically in cloud manufacturing, with the benefits of tamperproof recording in the decentralized or remotely operated processes [4]. This will support the smart systems in mass-producing highly customized products that require end-to-end integration of different autonomous manufacturing operations [5]. Additionally, intelligent robotics is undertaking delicate manufacturing tasks without human assistance [6]. This is evident from the advent of Industry 4.0 (I4.0), where the machinery development is accelerating towards the integration of software with multiple sensors in a smart factory environment [7], where the focus of conventional manufacturing is shifting towards the incorporation of decision-making capabilities [8]. This self-organizing manufacturing system consists of autonomous elements (e.g., software tools, equipment and operators) connected in a dynamic environment to perform in an unforeseen condition [9]. Therefore, Industry 4.0 (I4.0) defines a new technological era [10] of the intelligent factory architecture that will change the manufacturing processes driven by smart systems for self-structuring and self- monitoring [11]. Integration of these systems enables an intelligent manufacturing environment to yield productivity for high-quality customized products.

Cloud manufacturing (CM) can be adopted to overcome the challenges associated with the traditional systems for on-demand and reliable manufacturing capabilities [12]. To realize the smart factory [13], intelligent systems will require to replace human decisions, where production will be controlled autonomously and dynamically with a high degree of automation [14]. Thus, Industry 4.0 focuses on the subtractive, additive and hybrid techniques to connect with the cyber/digital realm. In this regard, a cloud-based framework was developed for online diagnosis architecture [15], where the machine tool and sensor system are connected to the cloud to coordinate and control the operation in a cyber physical system (CPS). Moreover, the smart monitoring can be implemented to reduce the risk of damaging workpieces, cutting tools and the machine itself by linking the physical resources (machines, tools, workpieces) with complex CPS with the use of sensors and IoT for the development of smart machining operations to increase productivity [16]. Additionally, an image-based system is considered to predict the tool wear relating to the surface quality of the machined parts with varying cutting conditions in subtractive manufacturing (SM) [17]. A digital twin (DT) driven process parameter optimization and surface roughness prediction method is proposed for real-time machining process monitoring [18]. Intelligent machining tries to enable intelligent behavior in the machining system [19] to avoid defects from human errors as, machines learn and adapt to optimize machining processes. Thus, the emergence of digital twin (DT) technology provides an opportunity to incorporate digitization and intelligence in materials processing technology. Additionally, the adoption of digital technologies can shift the paradigm across all manufacturing sectors [20]. In fact, several innovations have enriched additive manufacturing (AM) in various dimensions, including smarter materials and functional structures, smart and agile manufacturing ecosystems capable of traditional manufacturing [21].

Additive manufacturing (AM), commonly known as 3D printing, is the layer-wise manufacture of parts printed to the required shape [22] rather than wastefully cutting away material from a solid block of metal, ceramic, polymer or composite material. AM technology is capable of rapid prototyping, mass customization and decentralized production with the networking potential of connecting the vast number of machines concurrently [20]. By incorporating a ‘smart’ component into AM, a cyber physical system (CPS) can be developed to respond to market demand in real-time situations, thereby enabling the digital value chain [23]. While data-driven approaches in AM processing have made progress in the past decades, challenges remain in optimizing the process parameters to push forward for intelligent AM [24]. Thus, qualitative uncertainties become a key challenge for the further industrialization of AM techniques [25].

Though AM is gaining in popularity for making parts with complex geometries, however, the defects inherent to the AM-produced parts make it inferior to the similar part produced by conventional processes [26]. Therefore, various post-process techniques are employed to meet the part tolerances and surface quality requirements [27]. Nonetheless, some post-processing methods are time-consuming and not viable for complicated structures and/or significantly large surface areas [28]. To overcome this limitation, hybrid manufacturing (HM) processes are used [29]. Furthermore, hybrid manufacturing techniques can be applied to fabricate parts with complex geometry, where the manufacturing process is carried out with a combination of additive and subtractive manufacturing [30]. These processes can take place either concurrently or in sequence to complete the required task and there is no limitation on the number of processes utilized to produce the complex 3D part. More importantly, in the era of the fourth industrial revolution, hybrid processes progressed from the developments in information technology to enhance product quality [31]. Additionally, HM provides secondary operation to improve part quality after releasing residual stress originating from the AM process [32]. Again, AM parts require SM techniques (CNC machining) to ensure geometric tolerance and removal of support materials for which an integrated HM workstation enables such process much more easily and quickly [33]. Therefore, HM technologies leverage AM’s strengths for complex geometries and SM’s precision for finished parts [34]. However, a dynamic process plan is required for HM where online process control with intelligent characteristics as well as the feedback from the inspection is deployed [35]. Thus, emphasis is given to flourish the techniques for deposition, material removal and hybridizing processes through smart toolpaths, where measurement techniques play an important role in the quality control [36]. Hence, challenges relating to software integration prevail for the concept of smart manufacturing in real-time scenarios, where artificial intelligence (AI) can solve problems [37]. The advantages and disadvantages of the subtractive, additive and hybrid processes are listed in Table 1.

Artificial intelligence (AI), in contrast to natural intelligence, is the reasoning exhibited by machines and software [70]. Thus, both subtractive and additive technologies are compatible with automation, while improvements are needed for connectivity, data sensing and collection [71]. Moreover, AI-driven image analysis can be used in post-manufacturing component inspections [72], whereas the data-driven approach explores correlations between input and output without explicit physical interpretation in real-time diagnosis [73]. AI works by developing intelligent agents to take actions by observing the environment to successfully achieve the predefined goals [74]. Therefore, prospects of intelligent manufacturing include remote real-time detection and control, defectless manufacturing through process planning and scheduling and predictive maintenance of machine tools/equipment. Moreover, in digital manufacturing, the automation is incorporated to realize intelligent transformation in a knowledge-evolutionary device [75] that can make decisions in machine to machine (M2M) communication environment [76]. Hence, a rule-based system (for subtractive manufacturing) was implemented to enhance the intelligence of machine tools to calculate and determine relevant cutting parameters [77]. Additionally, autonomous path planning and in-situ parameter tuning can be realized (for additive manufacturing) through online identification and feedback [78]. However, most of the existing control systems execute a fixed program, and hence, control of the functions happening outside the module has inflexible interfaces that cannot communicate unknown information [79]. Therefore, the purpose of this article is to fill this gap by giving researchers and engineers directions for intelligent applications of new technology across all manufacturing divisions, including AM or 3D printing process. The structure of the remaining article consists of Section 2 describing the literature review process; Section 3 describing the intelligence in manufacturing; Section 4 on the state-of-the-art manufacturing technologies; Section 5 about the critical analysis and challenges; Section 6 presents the prospects and Section 7 describes the conclusions.

## 2. Review Methodology

The prospect of this article lies in the comprehensive review of the literature to establish the status of intelligence utilized for subtractive and additive processes and their sustainable merger. Various research works have been reported in the past on related technologies and their fundamental challenges. However, the current review emphasizes the present and future scenarios of the application of intelligence in additive and subtractive manufacturing within the context of Industry 4.0, emphasizing automation, artificial intelligence, process monitoring, sensorial systems, human-to-machine and machine-to-machine interface.

To analyze the present and future scenarios concerning the progress of traditional additive and subtractive manufacturing to intelligent additive and subtractive manufacturing, the systematic literature review (SLR) is applied, which is different from the narrative literature reviews. The focus of SLR is to evaluate the collected evidence against the predetermined criteria by minimizing the bias in the study selection. The review begins with a focus on research agendas. To explore the research agendas, the SLR framework for this study is followed from the literature [80,81]. While planning the review, the research question (RQ) is fixed to present the implication of intelligent additive and subtractive manufacturing based on the research scope, as per the following:

RQ1. What is the role of intelligence in additive and subtractive manufacturing?
RQ2. What are the major challenges of intelligence in additive and subtractive manufacturing?
RQ3. What are the present trends of intelligence in additive and subtractive manufacturing?
RQ4. How to prepare the present intelligence of additive and subtractive manufacturing for the future?
RQ5. What future directions need to be followed from the status of intelligent additive and subtractive manufacturing?

The RQ1 and RQ2 focus on the functionality of intelligence in additive and subtractive manufacturing. RQ3 is concerned with the present scenario of the study objective from the conventional state and new interventions in this field. RQ4 and RQ5 address the challenges, scope and impediments of the current technologies towards the future. An overview of the present and required technologies for the future, alongside the challenges and limitations that have occurred to integrate with the field are discussed to answer the questions. Once the research question is defined, the SLR moves to the search phase. The search work mainly finds the review article, journals and conference proceedings that are significant and aligned with Industry 4.0 technologies with context of intelligence in AM and SM amalgamation.

**Figure 2:**
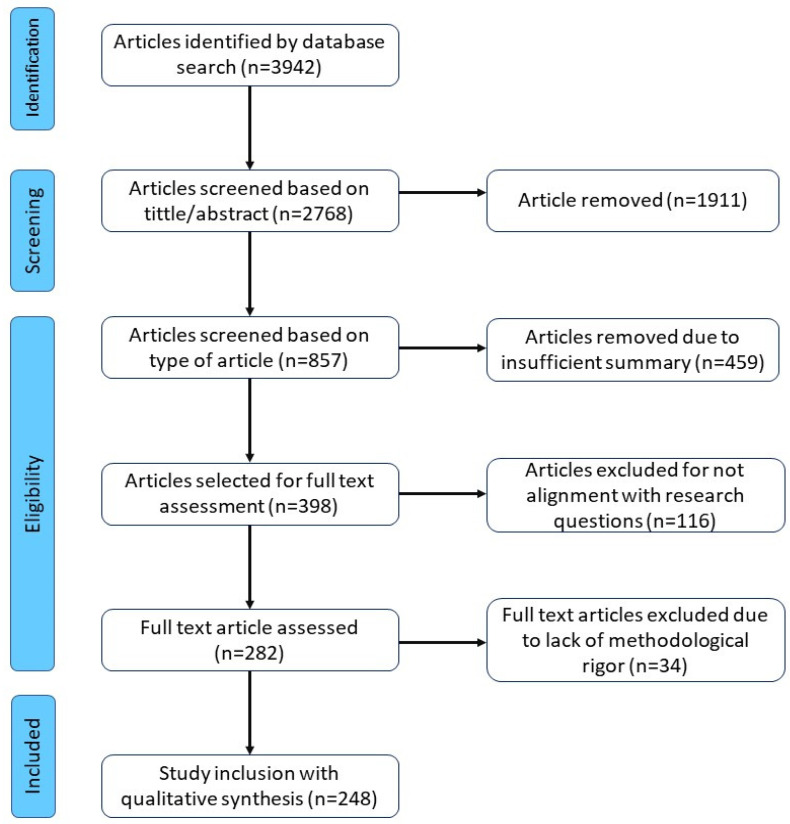
Numeric flow diagram for PRISMA regarding the literature review.

The focus of the review is targeted at specific keywords due to the high-volume availability of content. The search is conducted in scientific databases such, as Google Scholar and Science Direct. Google Scholar provides the information about the journal name, and publication year and the advanced search option helps to cover citations that are not covered by other databases. The search strings for the publication search are the following: ‘Intelligent’ OR ‘Smart’ OR ‘Intelligence’ AND ‘3D printing’ OR ‘Additive manufacturing’ OR ‘Direct manufacturing’ OR ‘Three-dimensional printing’ OR ‘Rapid prototyping’ OR ‘Digital manufacturing’ OR ‘Generative manufacturing’ OR ‘Additive fabrication’ OR ‘Solid free form fabrication’ OR ‘Rapid manufacturing’. About 3942 publications were found in the timeline 2005–2022, based on the search string on 31 December 2022. Then, the title of the articles were screened to remove duplicate articles and/or articles not related to this research, thereby 2768 articles were sustained. Various types of grey literature, presentation, keynotes and inaccessible publications were excluded, and the number of articles was reduced to 857. Then, the abstracts were read for subsequent evaluation to obtain the research question answers. About 398 articles were selected for full abstract reading synthesis to cover the review work. Then, 116 articles were removed for not aligning with the research questions and 282 articles went through the full text assessment. Finally, 248 articles were selected for qualitative synthesis for reviewing in the literature. The transparency of the results produced by the searching process included in a systematic review is thus highlighted by the risk of bias evaluation. A flowchart outlining the PRISMA algorithm focuses on the reporting of reviews, evaluating the effects of interventions and serves as a foundation for reporting systematic reviews in numerical form is illustrated in Figure 2.

**Figure 3:**
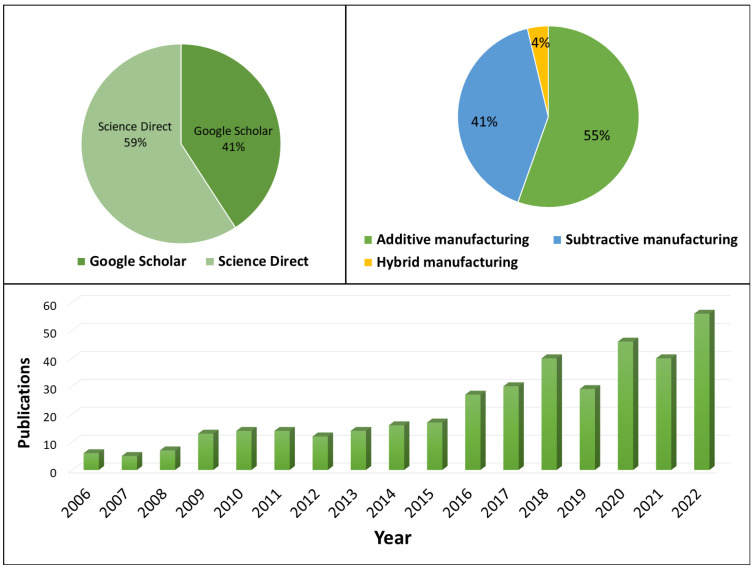
Visual representation of the article’s percentage according to the search engine (**upper left**), manufacturing process related proportion (**upper right**), and article’s year growth (**lower portion**).

From the article qualitative synthesis, the present state-of-the-art intelligence in additive, subtractive as well as hybrid manufacturing has been produced. Moreover, the challenges and research gaps for the existing state for future expansion of intelligence related to manufacturing connectivity, in-situ monitoring systems, human to machine interference, machine learning application in the sensorial system, artificial intelligence in decision making, collaboration among robots, machine to machine cognitive, digitation and adaptability have been identified. The search engine papers percentage, proportion of AM and SM, hybrid manufacturing articles review and year growth of publications are summarized in Figure 3.

## 3. Intelligent/Smart Systems- Definition, Principle, Prerequisites

**Figure 4:**
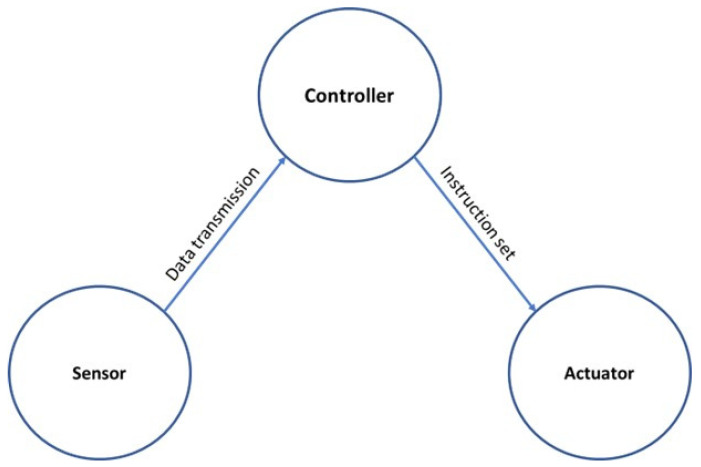
Schematic representation of the basic architecture of a smart system.

The notion of the intelligent/smart system emerged from an area of research and development that envisaged a system consisting of various sensors, controllers, and advanced materials to mimic biological mechanisms [82]. The basic component and arrangement of the smart system is shown in Figure 4, where multiple sensors collect data from the environment and send those to the controller through the process of data acquisition. The use of a multitude of sensory data is very common in a smart system termed sensor fusion. Once the controller receives all the data, it uses various algorithms to control the actuator, ranging from simple feedback control to intelligent control. The smart home is an example of an intelligent system that researchers and industrial players have thoroughly investigated. Smart homes consist of the essential components (Figure 4) of the smart system to control the ambient condition of the building, such as lighting and heating. However, the concept of the smart home has evolved, and now smart home indicates data-driven intelligent control of any electrical component within the house [83].

Like the smart house, smart manufacturing is an overwhelming term used to describe the futuristic industry. The advancement and modularization of computers and electronics-initiated automation in manufacturing, is known as the modern manufacturing era. Nowadays, machine tools, critical components of manufacturing, are operated mainly by the CNC (computer numerical control) system with minimum human intervention. As with the machines, raw materials are also handled by automated conveyors or AGVs (automated guided vehicles) and stored in ASRSs (automated storage and retrieval systems). Automated manufacturing can be categorized in various ways depending on the scope and degrees of automation and intelligence in manufacturing [84]. Two significant aspects of manufacturing are subtractive and additive manufacturing. Subtractive manufacturing, in other words, machining, also has recently become highly automated and intelligent.

Smart/intelligent machining systems are aimed to have the capacities of self-recognition with the abilities of self-monitoring and optimization of operations; self-assessment of the quality of work; and self-learning for improved performance over time [85] through the synergic assimilation of hardware and software. Moreover, AM has increasingly become more popular for rapid prototyping. However, AM still has a long way to develop critical functional parts due to its inherent nature of layered structures. In most cases, functional parts with tight tolerance and strict surface integrity cannot be achieved by using AM technologies alone. Therefore, AM manufactured parts commonly require some post-processing, such as machining to meet the requirements associated with high surface finish and dimensional tolerances. Moreover, application of smart technology is also quite evident in the field of AM, especially for the in-situ assessment of the parts and quality control by applying feedback control of the process parameters. The application of AI and ML to better the AM process is another growing research area.

## 4. Intelligence in State-of-the-Art Manufacturing Technology: Monitoring, Feedback, Controlling, and Machine Learning

### 4.1. Subtractive Manufacturing/Machining

**Figure 5:**
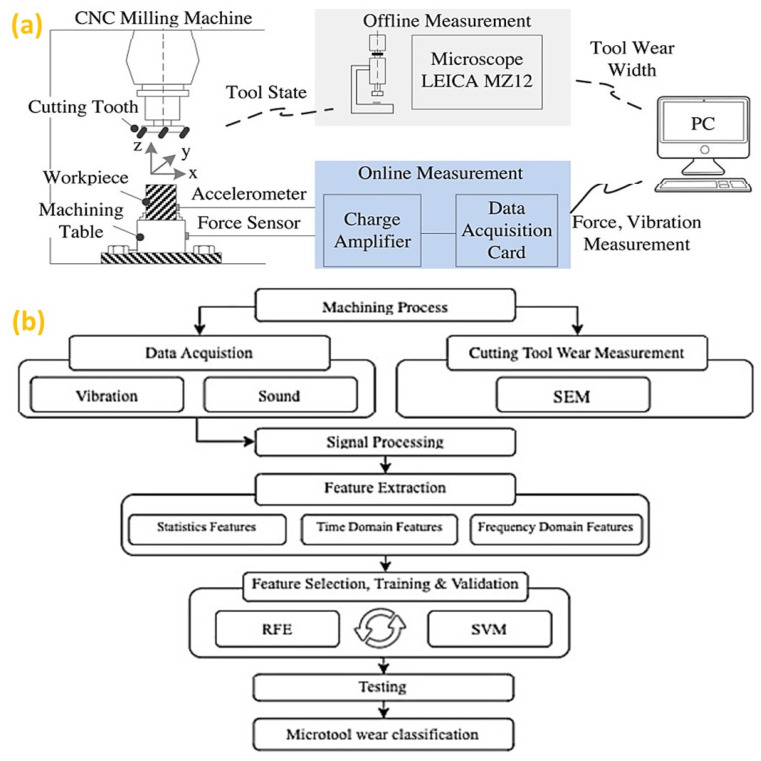
Visual schematic diagram of (**a**) the experimental setup (reprinted with permission from [89]. Copyright 2020 The Society of Manufacturing Engineers). (**b**) Framework for tool wear prediction (reprinted with permission from [91]. Copyright 2020 Elsevier B.V.).

From an intelligent manufacturing context, the research focus has been directed toward intelligent machining [86] with an aim for online monitoring and optimization of the machining parameters [87]. Thus, monitoring the machining state (i.e., tool condition, tool wear, machine stability, chatter, and vibration) becomes an important research area for intelligent subtractive manufacturing. Additionally, the prediction of tool wear is attracting attention to improve the part quality by reducing the scrap rates, thereby enhancing the productivity and sustainability of manufacturing operations [88]. The life cycle of the tool can be predicted from the offline measurement of tool flank wear, corresponding to the multi-sensor data collected online during a high-speed CNC machining experiment, as shown in Figure 5a [89]. Integrating physical knowledge with a data-driven model helps tool wear observation in a dynamic condition [90]. Figure 5b illustrates the framework to monitor tool wear in micro-milling processes [91]. The method first started by acquiring vibration and sound signals and then, by using scanning electron microscopy (SEM) images to detect the tool wear. Subsequently, the most significant features are selected by a recursive feature elimination (RFE) method from the various features related to statistical analysis, time domain and frequency. Once the features were selected, a support vector machine (SVM) model was created to predict tool wear. One of the shortcomings of this method is the off-line tool wear measurement by superimposing SEM images of the new and used tools.

**Figure 6:**
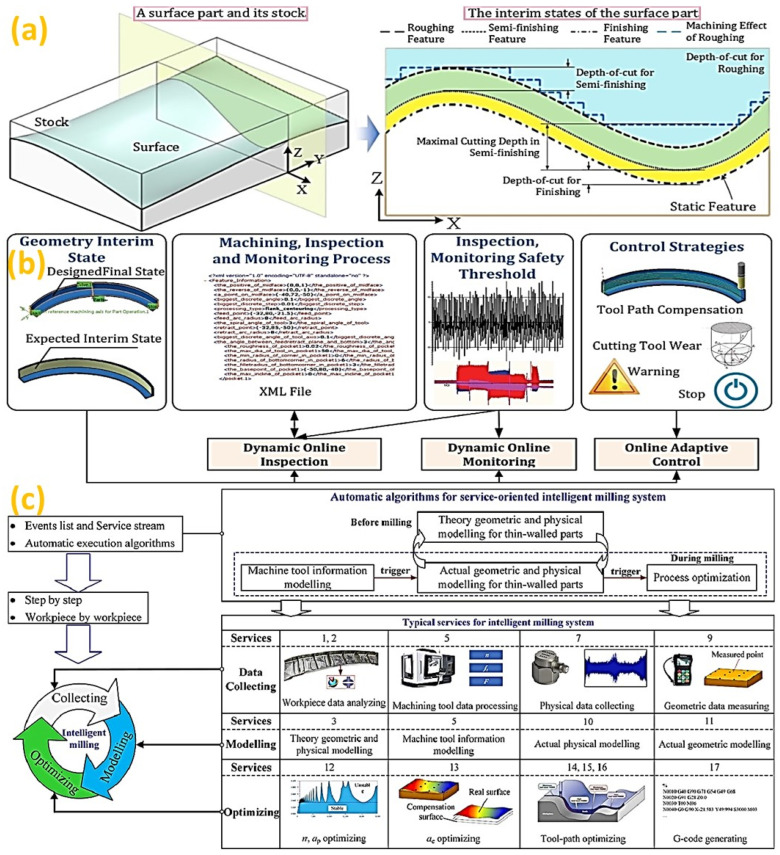
(**a**) Various depth-of-cuts in machining a freeform surface (reprinted with permission from [93]. Copyright 2015 Springer Nature). (**b**) Dynamic feature information and its applications (reprinted with permission from [94]. Copyright 2015 IOS Press). (**c**) Three parts of an intelligent milling system, including data collection, information modelling and process optimization (reprinted with permission from [95]. Copyright 2022 Elsevier B.V.).

For complex parts machining, the dynamic feature representation method is utilized where features are extracted from the CAD model [92]. This contrasts with the traditional process where modelling is conducted on the static feature of final geometry, which is assumed to be unchanged as the machining progresses. Hence, it is necessary for the representation of interim features corresponding to planned depth-of cuts, as illustrated in Figure 6a [93]. In this respect, the dynamic feature concept is utilized for machining process optimization, as illustrated in Figure 6b [94]. By collecting in-process geometry information of the workpiece, a data-driven information model is constructed based on the actual cutting condition for adaptive machining process optimization. Therefore, intelligent machining can be executed as an automatic process, as illustrated in Figure 6c [95]. Hence, it is necessary to inspect the workpiece conditions for defect detection and to control the machining strategies in response to workpiece dynamic conditions in an intelligent machining system.

**Figure 7:**
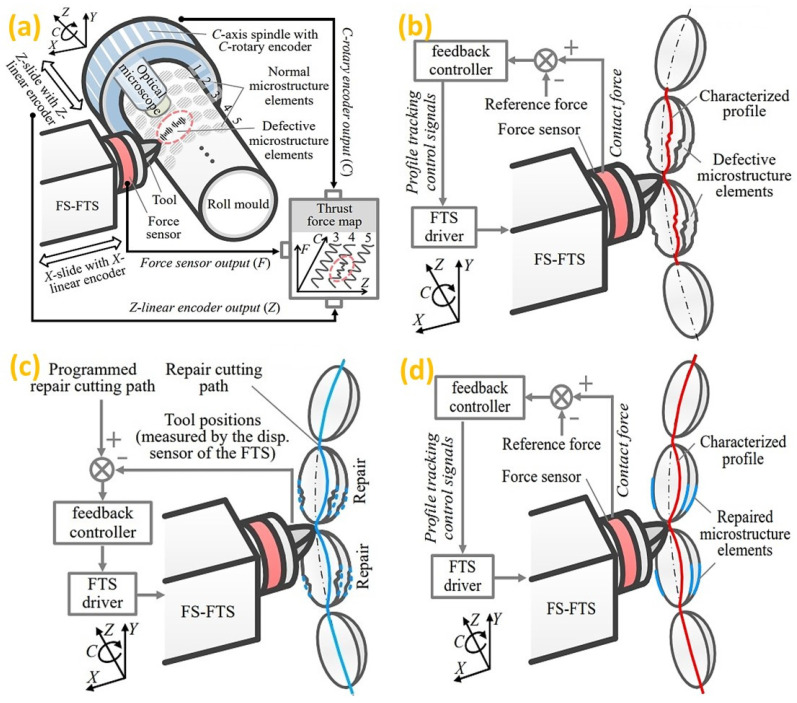
In-process measurement, repair, and evaluation method of defective microstructures on a roll mold illustrated as (**a**) the real-time detection of the defect positions; (**b**) characterization of the defect surface profiles; (**c**) repairing the defective microstructure elements; and (**d**) evaluating the repair results (reprinted with permission from [97]. Copyright 2014 Elsevier B.V.).

In the recent past, ultra-precision machined parts have been increasingly used in various emerging fields. However, most machining aspects depend on the operator’s skills for optimizing the parameters by costly trial-and-error experiments [96]. To address these issues, an in-process measurement technique was utilized to repair defective workpieces on a roll mold machined in an ultra-precision lathe [97]. As illustrated in Figure 7, the method works when a force feedback control loop is applied to guide the cutting tool. Therefore, the feedback of the measured information is used for generating an accurate repair cutting path. By replacing the conventional post-manufacturing inspection made on an off-machine, on-machine surface metrology is utilized for the task of compensation machining, feedback process and machine tool diagnosis [98]. As one of the future trends, on-machine measurement and monitoring will be an essential part of the advanced machining process for automatic compensation tool path generation to realize the IoT-based activity in intelligent manufacturing.

**Figure 8:**
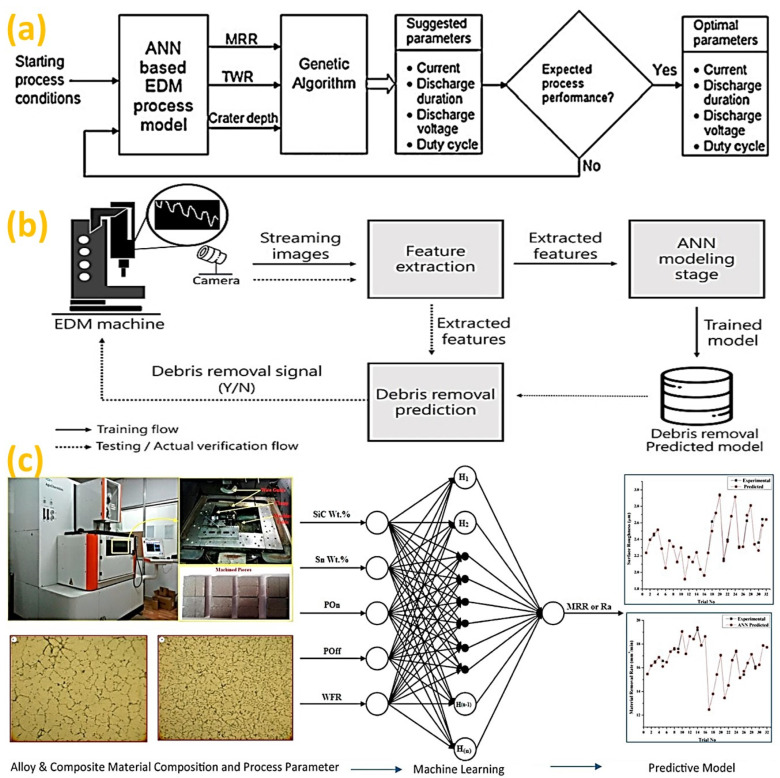
(**a**) integrated ANN–GA approach for process optimization (reprinted with permission from [101]. Copyright 2010 Elsevier B.V.); (**b**) intelligent system framework of debris removal operations [105]; (**c**) machine earning (ML) and predictive modelling for WEDM process (reprinted with permission from [107]. Copyright 2018 Elsevier B.V.).

In addition to conventional machining, machining conditions were predicted for non-conventional processes [99]. Electrical discharge machining (EDM), one of the unconventional machining processes using electric energy to remove materials, has been widely used in high-performance engineering sectors [100]. An intelligent approach utilized for the optimization of die-sinking EDM parameters is illustrated in Figure 8a [101], where physics-based process modeling was integrated with artificial neural networks (ANNs) and a genetic algorithm (GA) to predict the shape of a crater, material removal rate (MRR) and tool wear rate (TWR). In early research, an on-line monitoring approach was used [102] to establish the tool-workpiece gap signals correlation with pulse types in EDM. To enhance the performance of EDM, a hybrid electrical discharge and arc machining (HEDAM) module were developed [103]. Studies proposed an intelligent pulse classification method [104] and the efficiency improvement by real-time debris removal, as illustrated in Figure 8b [105]. To predict the surface roughness of aluminum alloys machined using wire electrical discharge machining (WEDM), multiple machine learning algorithms were used [106]. To collect data that were used to create the models, parts were machined using different machining parameters and subsequently surface roughness was measured. In another research, an intelligent approach and machine learning technique was used to create relationship mapping between WEDM parameters and output response, as illustrated in Figure 8c [107].

**Figure 9:**
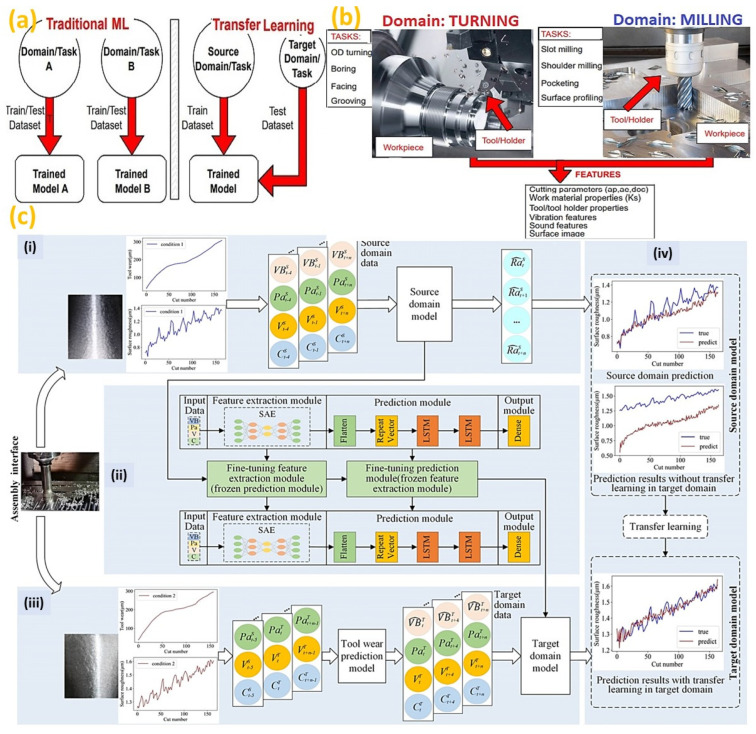
Illustration of the (**a**) concept of transfer learning; (**b**) domains, tasks and features of chatter detection (reprinted with permission from [112]. Copyright 2022 Elsevier B.V.). (**c**) Surface roughness prediction framework for the assembly interface: (**i**) pre-training of the surface roughness prediction model in the source domain; (**ii**) transfer learning for the modules of the source domain model; (**iii**) prediction of surface roughness in the target domain; and (**iv**) surface roughness [113].

ML, a subset of AI, provides the capacity to learn and improve the systems from experience and thus, is widely applied in different areas of manufacturing [108]. In recent years, ML has been found to be a useful tool for in-process tool condition monitoring [109], predicting self-induced vibrations and chatter in heavy-duty milling machines [110], by creating complex analytical models and simulations. The experimentation was carried out to predict chatter based on different machine positions and milling directions [111]. As the initial machining condition changes, the ML model needs to be revised according to the new data, and thus, broader applications of ML methods are hindered. Transfer learning (TL) methods help to minimize this problem by transferring knowledge from a source domain to a different but related domain, as depicted in Figure 9a,b [112]. TL is applied to the online prediction of surface roughness under different cutting conditions, as illustrated in Figure 9c [113]. However, limitations exist in automatic labeling data to the target the domain model. Additionally, a hybrid method combining numerical modeling, cutting dynamics and an artificial intelligence surface roughness prediction model is notable for future research.

### 4.2. Additive Manufacturing/3D Printing

Additive manufacturing (AM) process is used to create complex shaped parts with very little material waste without additional expensive tooling or complex assembly. AM processes have been classified into seven categories according to ASTM [114], which are displayed in Table 2.

**Figure 10:**
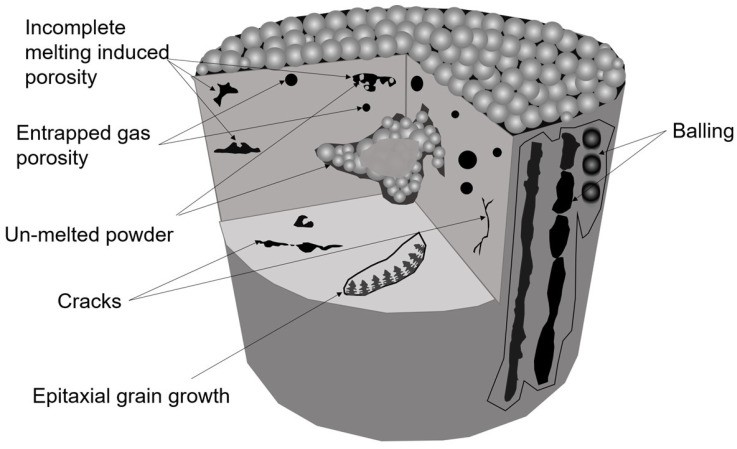
Defects in AM parts by selective laser sintering processes.

Additive manufactured parts may experience geometric flaws [115] due to surface defects (such as balling, high surface roughness, surface deformation, such as warping and distortion) and sub-surface defects, such as porosity [116]. Cracking, delamination on deposited material and residual stress are the thorniest issues associated with the AM technologies [117] for the SLS process, as illustrated in Figure 10. The presence of unwanted defects becomes the major bottleneck for widespread implementation of additive manufacturing in industries. Moreover, the mechanical properties, quality and reliability of AM manufactured parts depend on: (i) the chamber conditions, such as temperature, pressure, oxygen concentration [118]; (ii) the build environment condition, such as vacuum, inert gas or ambient [119]; (iii) laser deposition parameters, such as laser power, laser scanning speed, scan line spacing, power layer thickness and laser pulse length [120]; (iv) material morphology, such as chemical compositions of powders, particle size/shape, powder porosity, impurity and powder size distribution [121]; (v) machine specification, such as nozzle aperture, nozzle orientation and number of nozzles. The combination of all of these process parameters is mainly responsible for melt pool geometry [40], which not only determines the geometry and quality of the deposited track, but also directly relates to the local microstructures, defect size, defect morphology and mechanical performance (ductility and fatigue strength, corrosion resistance) of the built part [122]. Therefore, the process monitoring of AM can provide an insight into the influence of various parameters and can help in improving the build structure.

Although detection of defects in post fabrication offers insights into what might have gone wrong during the process, it is not viable for mass production. Hence, in-situ process monitoring of additive manufacturing is gaining significant momentum. Several metal AM processes are being used in industry, as well as in academia, however, in this paper, the powder-based fusion (PBF) process is given attention. The first step towards monitoring any process involves the use of suitable sensing devices. Different types of sensors have been employed for monitoring the additive manufacturing processes. The use of various techniques is aptly summarized [123] in Table 3.

**Figure 11:**
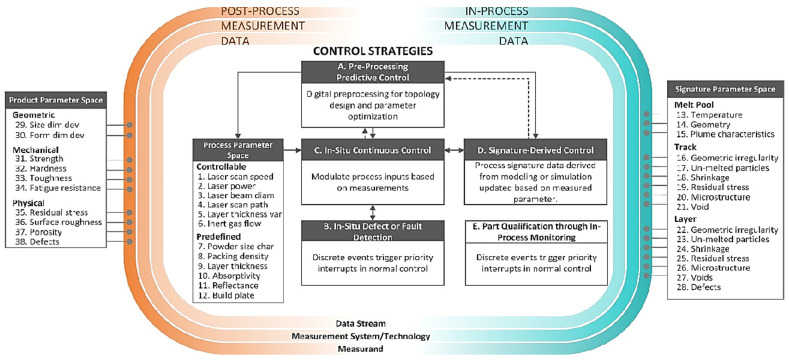
Defects in the proposed framework for the control strategies [144].

Most of the surface and subsurface defects are directly or indirectly caused by the overheating and uneven thermal distribution. Although several techniques have been explored for the in-situ monitoring of AM processes, vision-based techniques are widely employed due to ease of use and cost-effective hardware. Despite advancement in sensing capabilities, most of the AM processes are open loop. In one of the pioneering works, Mani et.al [143] categorized 38 parameters into three groups by establishing the hierarchy of process parameters, process characteristics and built qualities. Subsequently, a framework for the control strategies is proposed, as shown in Figure 11 [144].

**Figure 12:**
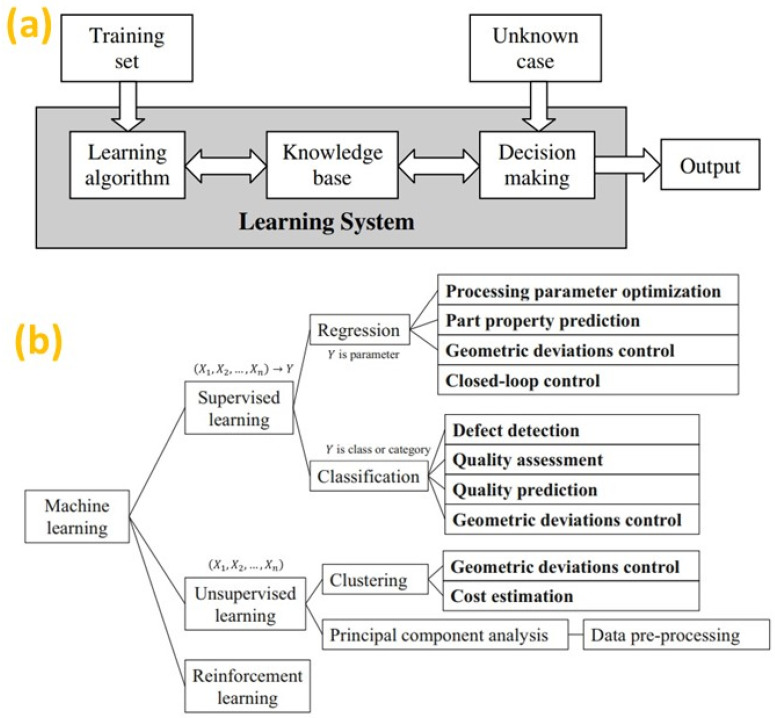
(**a**) General configuration of a machine learning system (reprinted with permission from [147]. Copyright 2004 Elsevier B.V.). (**b**) Taxonomy and applications of ML in AM as proposed (reprinted with permission from [148]. Copyright 2020 The Minerals, Metals & Materials Society).

Though significant work has been carried out in real-time fault or defect detection, more work is essential for the real-time process control. A sensor is deployed to monitor the melt-pool and control the laser power in-situ based on the feedback from the sensor [145]. In the study by Vlasea et.al [144], a signature derived control strategy resulted in improved dimension accuracy. However, the studies are still in the preliminary phases, and there is a significant scope for future research in this area. Additionally, the recent advances in sensor technologies have led to an unprecedented quantity of AM data with high dimensionality and complexity [146]. Manual screening of such a massive amount of data is not feasible, hence it requires automated methods, such as ML techniques, as shown in Figure 12a [147]. For this purpose, the AI solution can be a more suitable approach for in-situ process monitoring [148] because ML can recognize patterns and regularities in large datasets, and ML models can learn from data without explicit programming. Therefore, the amalgamation of ML algorithms and in-situ sensors can furnish an optimum solution for improving the quality, reliability, and repeatability of AM products. In the context of AM, ML techniques are broadly classified into three groups as supervised learning, unsupervised learning, and reinforced learning, as shown in Figure 12b [148].

**Figure 13:**
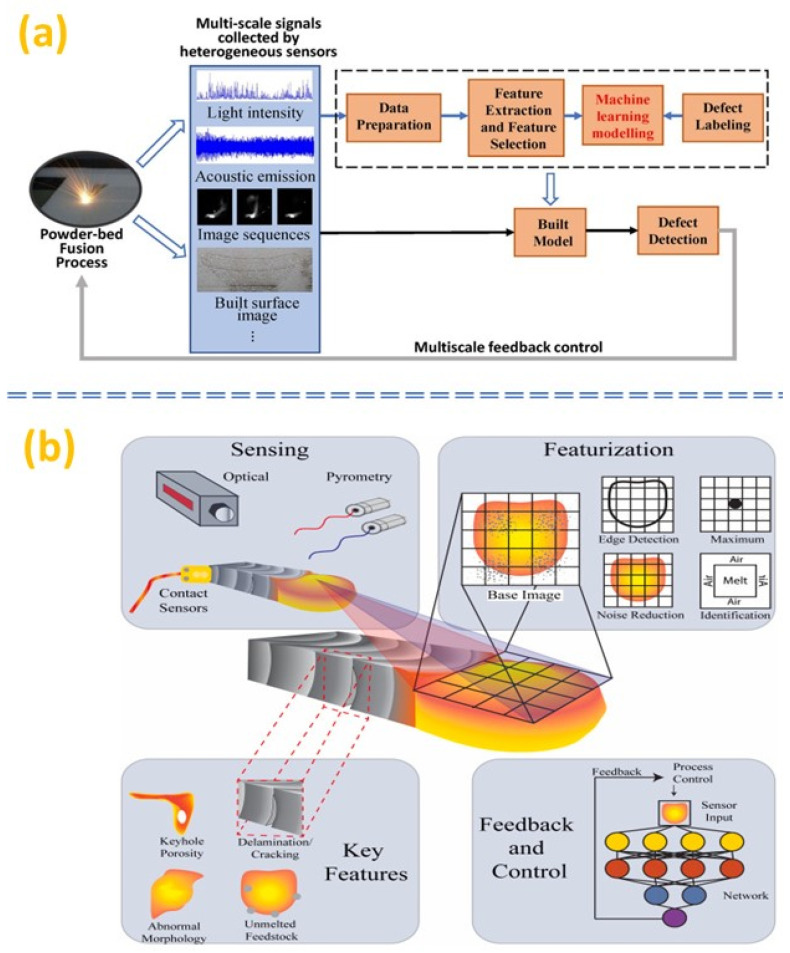
(**a**) Powder bed fusion (PBF) process monitoring and control framework (reprinted with permission from [151]. Copyright 2022 Springer Nature). (**b**) Multiple data sensors and feature detection for a wide range of signal monitoring, feedback, and control (reprinted with permission from [152]. Copyright 2020 Elsevier B.V.).

The additive manufacturing life cycle consists of five stages, namely: (i) design; (ii) process and performance optimization; (iii) in-situ process monitoring and control; (iv) post-process monitoring and control; and (v) testing and validation [146]. ML can be used in each stage to support new designers in the additive manufacturing design phase [149]. Moreover, faster and more accurate defect detection with ML techniques will drive the research efforts in this area. However, the unavailability of the common data format and standards remains a hurdle to overcome soon for the wide-scale adoption of the ML in AM applications. For instance, when a melt pool is present in the AM process, the visual information acquisition of the deposition area can be challenging due to the brightness and frequent sparkling, not to mention the evaluation of the defects thereafter. Therefore, emerging sensor techniques are heavily utilized and studied for AM processes, to provide both visual and non-visual information to better assist the in-situ monitoring process. Hence, intelligent detection technology utilizes on-site detection and intelligent algorithms combined with ML for image processing [150]. Thus, the sensing method, dataset preparation, feature selection and modelling algorithm are becoming focused along with the data fusion and feedback control, as shown in the framework illustrated in Figure 13a [151].

Additionally, following the recent trend towards the IoT-based Industry 4.0, closed-loop control systems and smart integrated devices are rising rapidly. Both academic research and industrial development are generating continuous new solutions, especially since progresses in ML and AI have enhanced the understanding of monitoring results of various AM processes even beyond the limit of human observations. ML or AI to assist in decoding the information acquainted has been a dominant trend in recent studies, which helps the system to identify microporosity [153], microcracking [154], melt pool conditions [155] and material feeding (powder bed) stabilities. By establishing a closed-loop feedback system, the acquainted information from monitoring can be directed back to the controller to improve the process in different ways. However, due to the complexity of the process parameters, from fluidic dynamics to thermal dynamics, the rapid solidification of melted material can make traditional process monitoring approaches difficult, thereby a major challenge becomes the prediction of the AM melt pool. Furthermore, there are various ways for monitoring the manufacturing process, as well as various types of sensors, as shown in Figure 13b [152]. Though acoustic and thermal sensing approaches can provide reliable data, the prediction is still challenging. This could be improved through ML and AI, with sufficient training data captured within a controlled environment. The sharing of datasets can be a promising trend for understanding such complex thermal processes across different platforms.

### 4.3. Post Processing for Additive Manufacturing

**Figure 14:**
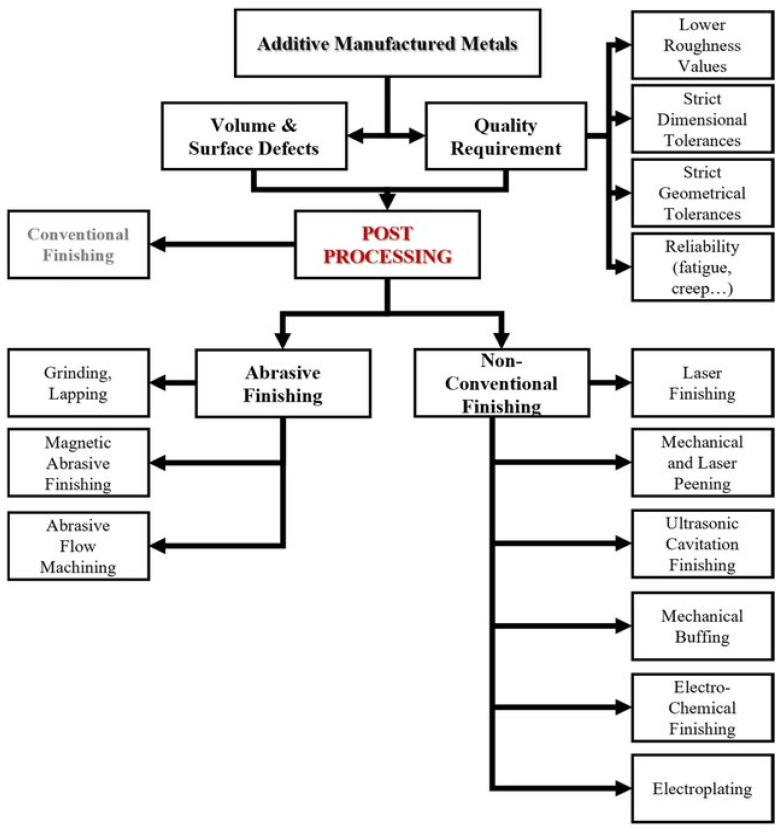
Various post-processing techniques utilized for metal AM (reprinted with permission from [156]. Copyright 2022 Springer Nature).

The online monitoring has become an inevitable part in product manufacturing, as it serves as an input for automating the whole process. Consequently, the production run time can be reduced significantly, which in turn reduces the production cost. The feedback received from online monitoring can be used for controlling the process using a trained algorithm model in soft computing, thereby complex nonlinear processes can be effectively modelled using such techniques. The entire automation platform includes sequential steps of monitoring, feedback, controlling and machine learning. The automation technique is well established in subtractive manufacturing and is gaining ambience for the post processing of additive manufacturing parts too. In general, post processing techniques have been classified, as shown in Figure 14, [156] with the three main subheadings derived from the energy used to create the polishing effect where the needs of post-processing techniques for AM metals increase [157].

**Figure 15:**
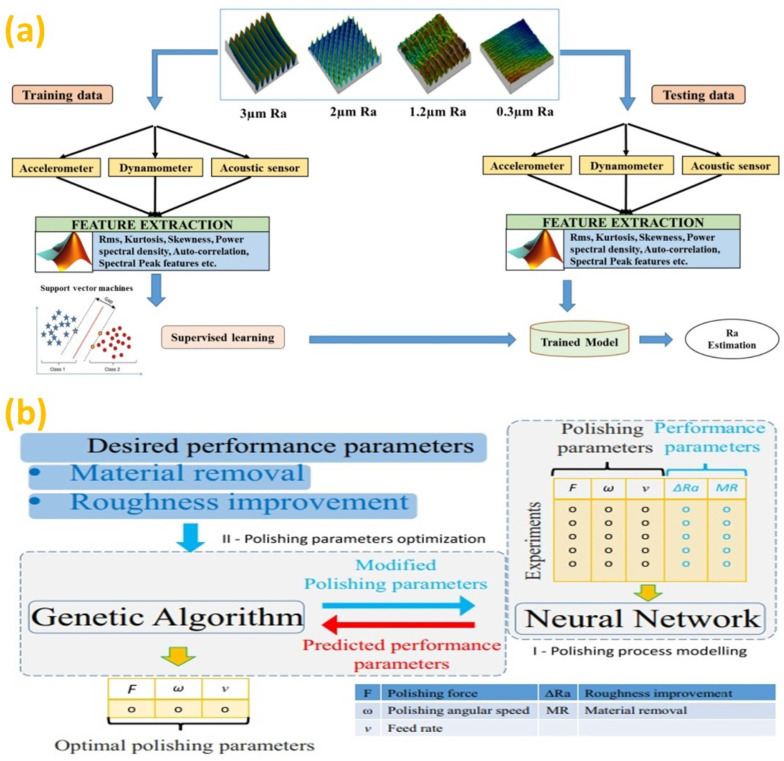
(**a**) Strategy for predicting surface roughness using the SVM classification algorithm (reprinted with permission from [162]. Copyright 2016 Elsevier B.V.); (**b**) Combined NNW and GA strategy for polishing uneven surfaces (reprinted with permission from [163]. Copyright 2017 Springer Nature).

An initial attempt of research in this area was performed on neural network modelling for abrasive flow machining (AFM) operation [158], intended to polish materials with an internal flow path. The generated model was then coupled with a heuristic search algorithm to choose the machine setup and process parameters required for the AFM process. In another study, surface finish was predicted for non-conventional electro-thermal processes, such as EDM [159]. The strongly influencing parameters, such as pulse current and pulse duration, as well as workpiece material were assigned as the input for the neural network developed. In due course of time, the artificial neural network (ANN) established its ambience in several finishing operations. For magnetic abrasive finishing (MAF) [160], in-process sensor monitoring was carried out and the signals of force, as well as acoustic emission were captured, analyzed and given as input to the ANN for predicting the surface finish. In addition to the ANN, modelling using fuzzy logic was also performing well in online monitoring and response prediction [161]. The relationship of the percentage improvement in roughness with input process parameters, such as voltage, rotational speed of the electromagnet, machining gap and abrasive size was referred for constructing the fuzzy interference system (FIS). In a later study, an in-process multi-sensor integration system was developed to capture the dynamic behavior of the abrasive belt machining process [162]. The system consisted of sensors capable of measuring surface roughness in real-time. Samples with different roughness values were subjected to abrasive belt machining under the same conditions. The features are extracted from the signals acquired during machining and are used to train the classification model using a supervised learning method based on SVM. Following the training of the model, a new set of signatures are generated for the same roughness values to analyze the model’s robust behavior. The proposed system can effectively predict the surface finish in a compliant abrasive belt machining process. The implementation of the whole technique can be understood from Figure 15a [162].

Nowadays, machine learning is gaining momentum with the aid of soft computing techniques and is moving forward to be an integral part of post-processing machining operations. The noteworthy aspect is that researchers have already started employing soft computing assisted machine learning techniques in the field of post-processing. An effective algorithm was developed for the conventional polishing method to eliminate the problem of non-uniform material removal on uneven surfaces during polishing [163]. Both NNW and GA were utilized for formulating the algorithm where the former was intended for process model generation, whereas the latter was meant for polishing parameters optimization. The proposed strategy can be interpreted from Figure 15b [163]. Initially, the polishing process was modelled through AI using a neural network. The results obtained from the experiments are then used to train NNW followed by testing with samples not used in the training stage. Then, the desired surface finish improvement and material removal are fed to the algorithm, which enforces GA to find the optimum polishing parameters. Therefore, machine learning regression methods were utilized for prediction of surface roughness [164].

**Figure 16:**
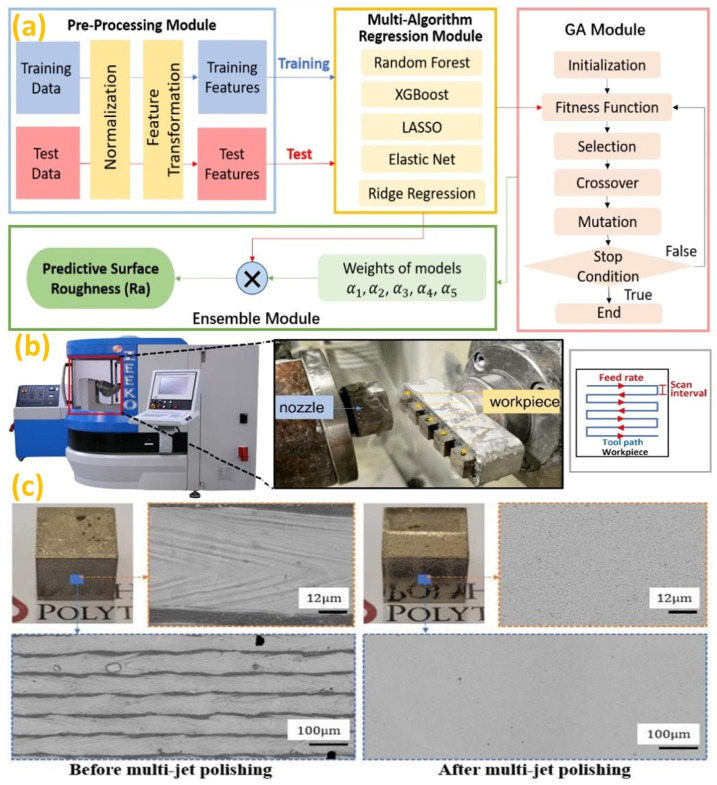
(**a**) Surface roughness prediction model framework. (**b**) MJP setup. (**c**) SEM images before and after MJP (reprinted with permission from [164]. Copyright 2022 Elsevier B.V.).

Artificial intelligence (AI) establishes its feasibility in even micro/nano-scale finishing operations. A recent study adopted artificial viral intelligence in abrasive flow nano-finishing process using the virus evolutionary genetic algorithm (VEGA) [165]. The study proved that VEGA could better adapt to the trends with experimental values in terms of surface finish. In recent times, image processing based on machine learning was deployed using convolutional neural networks (CNNs) to determine the laser polishing conditions responsible for contributing to better surface integrity [166]. Furthermore, electrochemical jet machining was utilized to finish AM parts created by PBF and to subsequently micro-pattern these for increasing part functionality [167]. In multi-jet polishing (MJP), a surface roughness prediction model was developed based on ensemble learning with a genetic algorithm (ELGA), as illustrated in Figure 16a–c [164].

Thus, the fusion of AI with AM and finishing/polishing methods can well control the automated post-processing of AM parts. Apart from including the input data for any finishing/polishing process during training, the data related to part fabrication using additive or subtractive techniques can also be incorporated to effectively predict the output responses of the post-processed component effectively. From the perspective of Industry 4.0, online monitoring and ML strategies are highly recommended for realizing reduced down time and minimal production cost. Correspondingly, the automated post-processing operations using ML and AI will pave the way for a novel research platform in the future.

### 4.4. Hybrid Manufacturing (HM)

**Figure 17:**
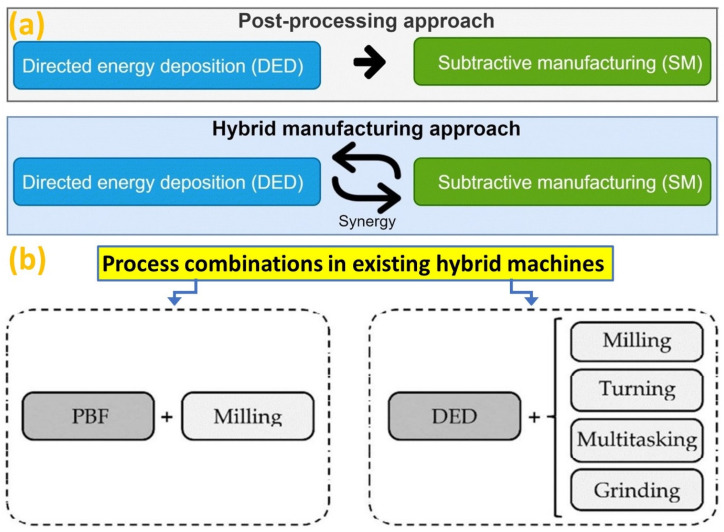
(**a**) Traditional post-processing and hybrid manufacturing approach (reprinted with permission from [36]. Copyright 2020 Springer Nature). (**b**) Different process combinations in hybrid machines [69].

Although traditional post-processing improves surface quality, conventional methods are not suitable for complex structures, especially for difficult-to-cut materials [28], thus, both the subtractive and transformative manufacturing technologies are combined with AM for improvement of part quality and process performance [168]. Therefore, the concept of hybridization [169] goes beyond post-processing, as illustrated in Figure 17a [36], where the hybrid-AM processes are usually cyclic processes involving synergy between both techniques, and are distinguished from postprocessing operations. Figure 17b shows the division of groups according to the primary additive technique [69] of PBF-based processes (directed to produce complex whole parts) and DED processes (focused on the generation of coatings). Thus, DED process can be combined with subtractive techniques and many machine tool builders are developing hybrid machines to overcome the AM drawbacks of low accuracy and high surface irregularities [170]. Combining multiple processes is advantageous to build an all-in-one hybrid machine [26] to make the best use of the strong points of each technology [100,171] with an aim of cost saving during manufacturing.

**Figure 18:**
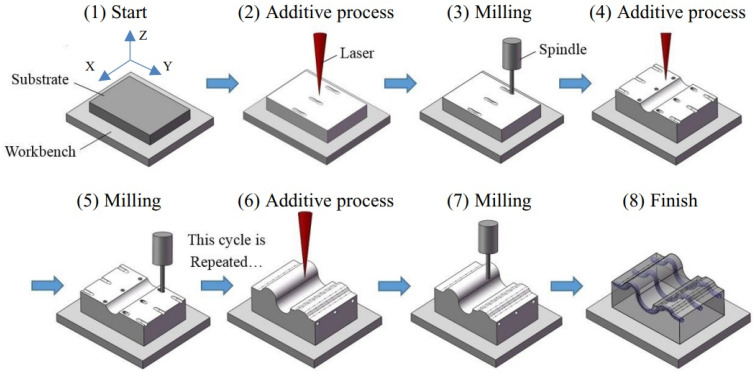
Schematic of hybrid additive and subtractive processes (Reprinted with permission from Ref. [26]. Copyright 2016 Elsevier B.V.).

Combining additive and subtractive techniques within a single workstation can achieve the benefits of hybrid manufacturing (HM) especially for remanufacturing of the high-value, custom-designed or discontinued components [33,100,171]. In addition, such HM process provides easy access to the secondary operations that improve part quality after residual stress is released from the AM process [32]. The process steps of HM are illustrated in Figure 18 [26], where the selective laser melting method is used to build the part from powders. Then, a milling cutter comes to machine the part, and after that, the additive process starts for the several successive layers. The additive and subtractive processes occur alternatively until the part is completed. While most of the academic research in HM is targeted for modelling and tool path strategies, a handful of industrial HM machines are available in the market. Table 4 lists industrial hybrid manufacturing systems.

**Figure 19:**
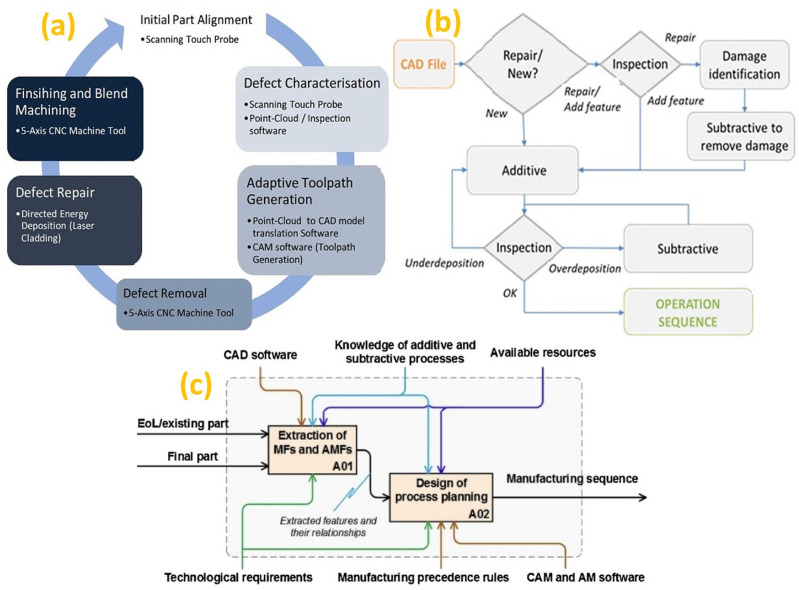
(**a**) Description of RECLAIM remanufacturing (reprinted with permission from [171]. Copyright 2015 Elsevier B.V.); (**b**) flow chart of the operation sequencing algorithm of the HM process [182]; (**c**) framework for the design of process planning (reprinted with permission from [183]. Copyright 2018 Elsevier B.V.).

To produce the desired part in HM, process planning is required for identifying the sequence of operations as the desirable outcome may be achieved by adding and subtracting material. Major steps of process planning identified for repairing/remanufacturing [180] include identification of the damaged feature, generation of machining, deposition and post-processing tool paths. To take advantage of both additive and subtractive manufacturing, combined additive, subtractive and inspection processes are gaining importance [181] in multi-purpose machine tools, as illustrated in Figure 19a [171]. Detailed description of the process sequence is illustrated in Figure 19b [182]. A two-step process planning is designed as illustrated in Figure 19c [183], where the technological requirements (tolerances, surface qualities) are obtained from the drawing. The features are identified and extracted in the first step (A01), thereafter, their relationships are used for process planning in the second step (A02). Thus, in hybrid additive manufacturing, CAM software is utilized to generate toolpath for additive, as well as machining processes [184] to set up the manufacturing sequence.

**Figure 20:**
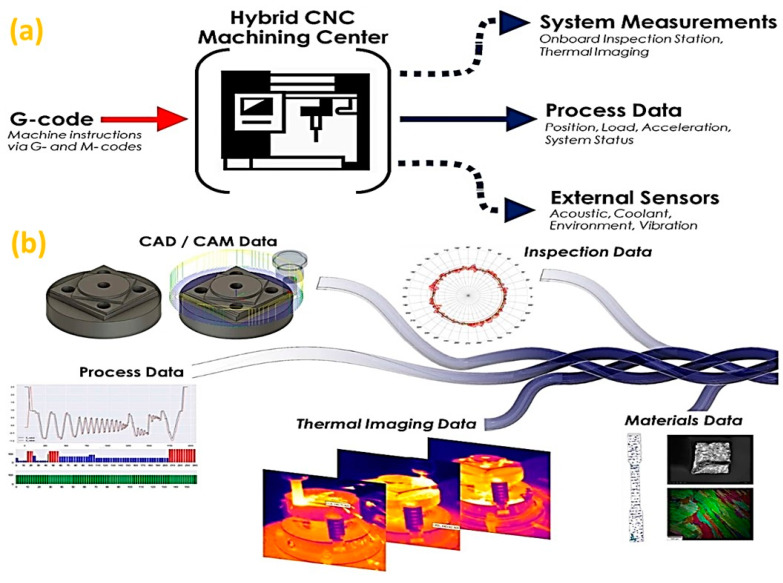
(**a**) Data streams in HM. (**b**) Spinning the digital thread with HM (reprinted with permission from [185]. Copyright 2021 Society of Manufacturing Engineers (SME)).

The operational instruction in HM comes from G-codes, in terms of toolpath trajectories generated from CAD/CAM software [185]. Moreover, open-platform standards (such as MTConnect) enable machine operational information to be accessible for monitoring [186], inspection and sensing data streams, as illustrated in Figure 20a [185]. However, the geometry data and the thermal history of a component are required for tool path planning for intermediate and final machining processes [184]. Thus, the construction of a digital thread [185] is demanded for collecting, synchronizing, fusing, and analyzing multiple data streams, inspection results and the thermal images captured during the operations. Moreover, machine operation and process information can be linked to a spatial location, while thermal images are collected and linked to a coordinated digital thread, as illustrated in Figure 20b [185] to enhance the development efforts of HM.

**Figure 21:**
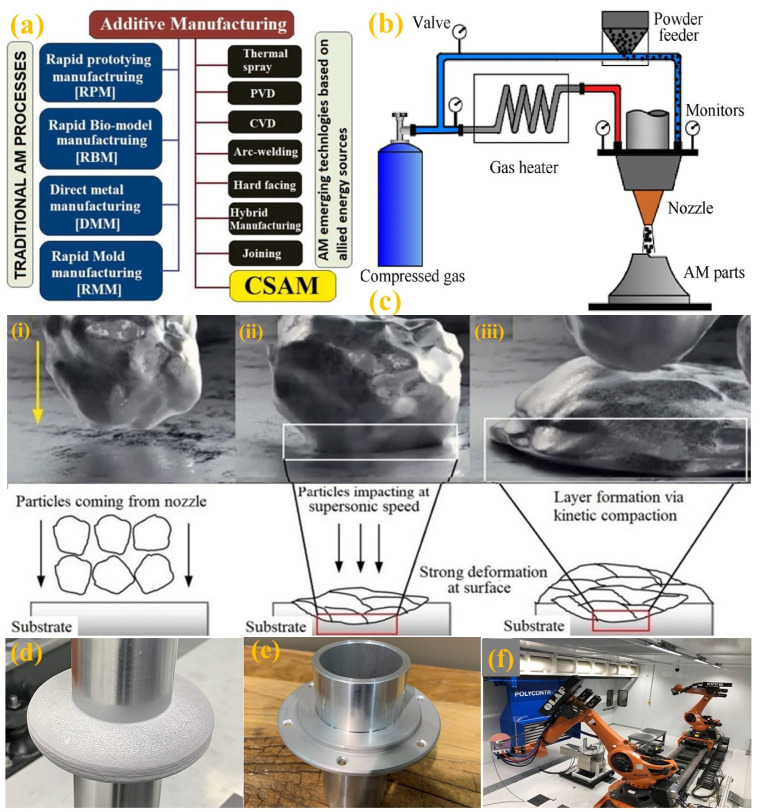
(**a**) Generalized AM techniques (reprinted with permission from [188]. Copyright 2021 Elsevier B.V.); (**b**) schematic of a typical high-pressure CSAM (reprinted with permission from [189]. Copyright 2021 Chinese Society of Aeronautics and Astronautics); (**c**) schematic diagram for coating development during CSAM (reprinted with permission from [188]. Copyright 2021 Elsevier B.V.); (**d**) pure Al on Al6061 (as sprayed); and (**e**) pure Al on Al6061 (after machining); (**f**) hybrid robotic cell (reprinted with permission from [190]. Copyright 2012–2023 Polycontrols Inc).

Integration of AM with the cold spray process—cold spray additive manufacturing (CSAM) is the solid-state supersonic deposition method that can build 3D components for mass production and remanufacturing [187]. Generalized AM techniques are presented in contrast to traditional AM methods, as shown in Figure 21a [188]. CSAM appears to be the most popular technique because it functions like the “3D printing” technique. Figure 21b [189] shows a typical CSAM system, where a fluidized powder mixture feed stock is fed into the gas upstream through the powder feeder before entering the nozzle. During the CSAM operation, high velocity powder particles impact on a surface, deform plastically and bond together to form a layer, as shown in Figure 21c. [188]. A noteworthy example of HM is PolyCSAM [190] which combines advanced surface preparation techniques, material deposition, in-situ robotic surface finishing, heat treatment and data analytics/machine learning-based process control. PolyCSAM’s integrated cold spraying processes with subtractive processes with a hybrid robotic cell is illustrated in Figure 21d pure Al on Al6061 (as sprayed), Figure 21e pure Al on Al6061 (after machining) and Figure 21f hybrid robotic cell.

## 5. Critical Analysis and Challenges in Intelligent Manufacturing

The application of additive and subtractive processes in industrial practices are rising rapidly [191]. AM processes are extensively used for various industrial, automotive, aerospace, communication, construction and medical applications, especially for highly customized and low volumes of production [192]. In the aerospace industry, AM has been utilized to fabricate products, such as rocket nozzles, turboprop stators, thrust chambers, cabin bracket connectors, bus structures, fuel nozzles and rocket engines [193,194]. Both AM and SM have been applied to produce a variety of custom medical implants in dentistry (teeth, crowns, bridges and dentures) [195], orthopedics (artificial limbs, knee joints and acetabular cups), cranio-maxillofacial (jaws and sculls) and devices (hearing aids) [196]. Recently, AM has been used to fabricate bridges for the construction industry [197]. Moreover, the components of electrical machines, such as shape profile windings, multi material coils, heating coils and hollow conductors were made by AM [198]. In automotive applications, AM is utilized for the fabrication of frames/chassis, suspensions, transfer cases, cross car beams, doors, and engine cradles [199]. Furthermore, hybrid manufacturing has added a new dimension to the advanced manufacturing process by addressing the drawbacks of both AM and SM processes to create more near-net forms, complicated, multilateral industrial components, such as metal molds [200], aero engine impellers [68] and sensor-integrated turbine blades [201]. However, the quality, surface integrity and reliability of HM produced parts is still a concern [202].

**Figure 22:**
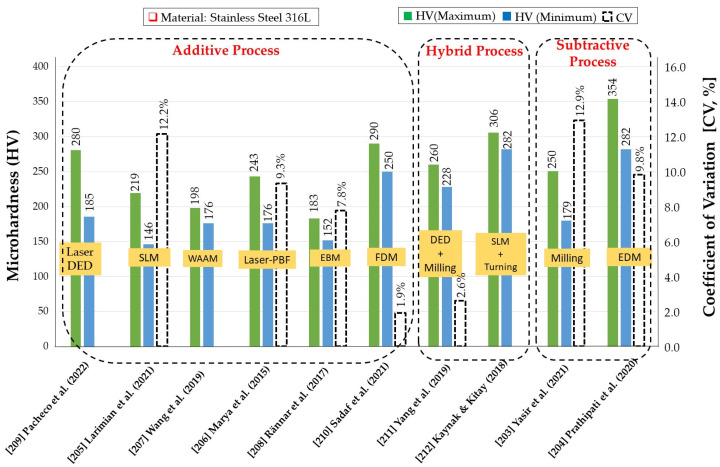
Microhardness variation of stainless steel 316L in different additive/subtractive/hybrid techniques.

Moreover, product property varies with the manufacturing processes and therefore becomes an industrial challenge. For example, the variation of microhardness of stainless steel 316L processed in AM, SM and HM techniques is displayed in Figure 22. Subtractive processes, such as milling, provide higher value of microhardness due to the high temperature generated at chip-tool interfaces [203]. Higher microhardness observed in the parts produced by EDM process due to the deposition of hard thin re-cast layer on the workpiece surface [204]. Comparatively lower HV values were observed due to the layer-by-layer building method by melting of the powder with a laser beam in SLM [205], fusion welding effect in laser-PBF processes [206], droplet-based deposition of melted metal wire in WAAM [207] and repetitive cycles of melting, solidification and annealing in EBM [208]. Generated residual stresses due to the thermal stresses result in a relatively higher hardness in laser-DED process [209]. The microhardness of FDM fabricated 316L, after post-treatment, such as debinding and sintering, are comparable to laser-DED fabricated products due to the influence of sintering temperature on the mechanical strength [210]. Again, the slightly lower hardness of the hybrid AM/SM fabricated 316L SS part is probably due to the coarser grain size and microstructure produced in DED+ milling [211]. On the contrary, finish-machining of SLM samples significantly increased the microhardness compared to as-built SLM samples due to the production of smaller grains and strain-hardened layers in SLM+ turning [212]. Figure 22 displays the consolidation of microhardness and coefficient of variation (CV) for stainless steel 316L in several AM/SM processes. Larger CV values (near 10%) in the subtractive process indicate a higher level of dispersion around the experimental mean values. Most additive processes show a similar level of CV except FDM, where CV values (below 2%) indicate reliable (consistent) measurements and good method performance. The variations of AM fabricated products due to porosity, inhomogeneous microstructure, solidification cracking, higher residual stress and distortion result in variations of microhardness. Hybrid process can reduce the variation, as observed from smaller CV values (below 5%) of microhardness in DED+ milling. However, it is not conclusive to assess the hybrid process with only one AM and SM process combination because multiple process combinations might cause different types of variation in microstructural characteristics. Furthermore, the number of available hybrid manufacturing machines is relatively low as technology is expanding. In this respect, extensive research is required by considering machine learning and digital twins to construct the link between physical property (microhardness) and part characteristics (defects) to make the process intelligent.

**Figure 23:**
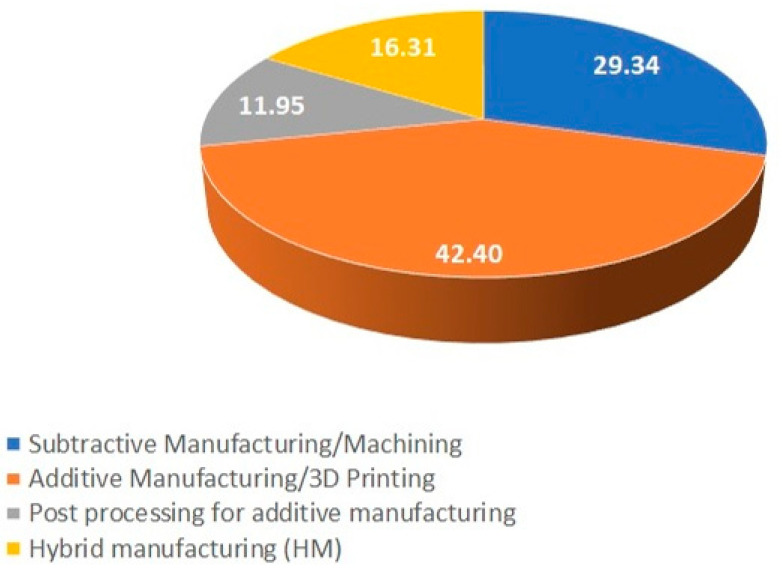
Analysis of the use of intelligence in different manufacturing technologies based on the literature survey.

Moreover, the analysis of research trends on the use of intelligence in various manufacturing technologies based on the literature survey is shown in Figure 23. It can be reasonably concluded from Figure 4 that the highest percentage of the use of intelligence is in additive manufacturing, which is more than 40% of the total research articles reviewed in this paper. This result indicates the significant growth of additive manufacturing in the research trend and the use of various smart features in this field. Interestingly, if postprocessing of the AM is included, then the percentage goes up to more than 50% of all articles.

**Figure 24:**
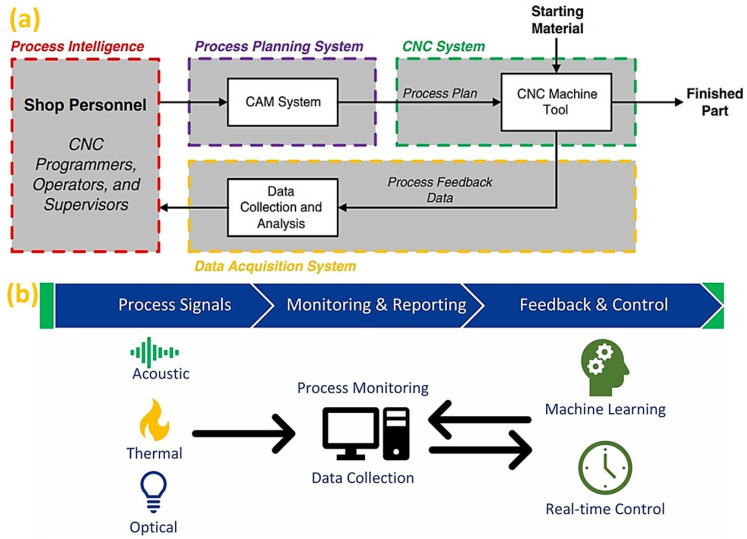
(**a**) Generalized traditional open-loop configuration of CAM and CNC systems with external data acquisition (reprinted with permission from [213] Copyright 2017 ASTM International). (**b**) Influence of process data monitoring with ML and real-time control in closed loop feedback [123].

In subtracting manufacturing, most CNC systems utilize an “open-loop” configuration; thus, require an integrated CAM/CNC system where data are input from process intelligence, as illustrated in Figure 24a [213]. Because of the separate realms in data acquisition and control, the self-optimizing smart CNC system is challenging. In some instances, changes made at the shopfloor cannot be directly fed back to the designer and thus shopfloor experiences cannot be preserved [214]. Therefore, the CAM system does not allow for automated process altering decisions based on real-time conditions. However, such a process plan improvement usually requires the CNC programmer to collect information from the process feedback data, and thus, requires significant manual effort. Moreover, machining process data collection is a difficult task as some control manufacturers do not provide provision for information collection and M2M communication [215]. This has put challenge on the data collection from multiple machines with different controllers and sharing that data into a common platform [216]. Furthermore, machine health condition data can provide significant insights into the real-time data analytics for ML algorithm deployment. Nonetheless, the CNC system should be aware of workpiece material properties to control cutting conditions and optimize the most effective toolpaths properly [217]. These requirements necessitate the development of an intelligent CNC system to respond to real-time (RT) process feedback, as well as machine tool operational status.

**Figure 25:**
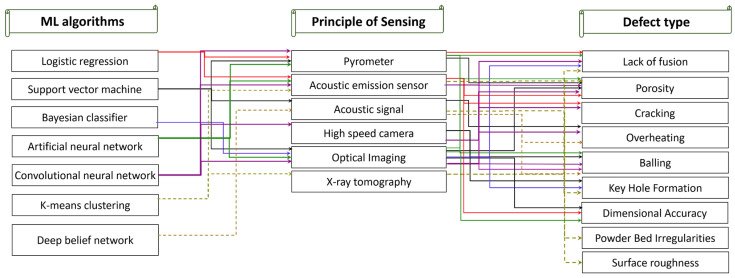
ML algorithms, sensing principles and detected defects in AM.

The inconsistent product quality becomes a major barrier to the widespread application of AM processes [146]. Hence, reduction of AM part variability have been recently given importance through in-process identification of material discontinuities and in-situ part inspection [218]. In-situ process monitoring of AM [219] can provide an insight into the influence of various process parameters. It can help improve the build process through closed-loop feedback control by detecting defects during the printing process with the help of sensors, as illustrated in Figure 24b [123]. By establishing a closed-loop feedback system, the acquainted information from monitoring can be directed back to the controller, to improve the process in different ways. Thus, the sensor technology should function properly in extreme conditions, such as in elevated temperatures. A significant challenge remains in real-time layer-wise defect detection and melt pool inspection with the high-speed cameras requiring high computational power [220]. Furthermore, predicting and altering the process parameters necessitate advanced ML algorithm techniques to integrate within the in-situ monitoring system capable of instantaneous feedback [221]. In relation to this, challenge remains on the structural health monitoring (SHM) system of the AM techniques [222] for microstructural characterization [223] of additively manufactured components. A list of commonly used ML algorithms, sensing principles and detected defects is shown in Figure 25. Choosing a suitable ML algorithm is crucial in achieving the appropriate level of defect detection in AM processes. For example, convolutional neural networks (CNNs) have emerged as state-of-the-art in terms of accuracy and robustness when dealing with image data. CNNs can also be used to detect various defects by using powder bed images, product layer wise optical images or melt pool images. Moreover, support vector machines with alternative kernel function are capable of handling both sensor signal data, as well as image data and is a good choice for classification. While implementation of an effective defect detection system is pivotal for the development of next generation intelligent AM technologies, the comprehensive understanding of the AM process and sensor accuracies need to be harmonized to handle different characteristics of the signal features.

**Figure 26:**
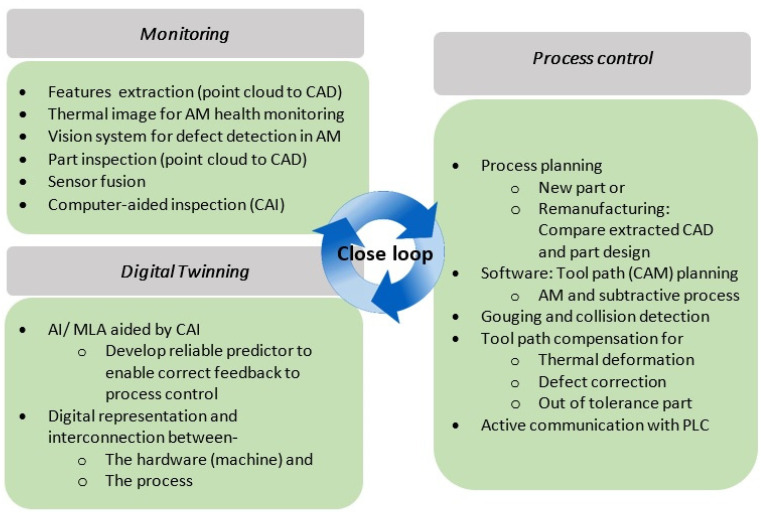
Essential features of intelligent HM post processing.

Current challenges related to online monitoring in post-processing reside in the large number of sensors required for precise data collection at a single instant, ultimately escalating the cost. Regarding machine learning, the performance of ML models is highly dependent on data analytics since highly accurate predictions require a huge collection of datasets for training the model. Moreover, similarity in captured surface features challenges model predictions using ML. For instance, melt pool in SLM can be recognized as spatters over the surface during image processing [224]. Although smoother surface finishing can be achieved by conventional polishing methods, complex structures cannot be processed using conventional methods [225]. Thus, research is directed towards novel post-processing methods to overcome the challenges. For instance, large-scale automation minimizes labor costs, as well as achieves superior finishing of freeform surfaces using a novel hybrid robot system [226]. Therefore, process intelligence will be the prime factor in the transformation of robotic hybrid manufacturing. However, the significant requirement for additional investment in hardware, controller capability and integration pose a challenge for online inspection and closed-loop manufacturing with auto-adjustments of the process parameters. The complexity of hybrid manufacturing remains when the available CAM software generates the toolpath strategies considering the gradient metallurgical properties throughout a component [184]. In this respect, the implementation of an NC-based digital thread can enhance the development efforts, including multi-part thermal monitoring, inspection-driven toolpath generation and in-situ laser modifications [185] to meet new possibilities of intelligent hybrid manufacturing [227]. Figure 26 shows the significant features that are essential to bring intelligence in HM post processing.

In summary, hybrid manufacturing (HM) approach has significant advantages over traditional post-processing with respect to geometrical complexity, difficult materials, accuracy, and surface integrity. Even though it is obvious that HM processing has advantages and has more flexibility with intermediate and final post processing for a compliant part; however, the technology has challenges to overcome. The challenges, their reasons and possible future direction intelligent (cyber) and physical HM post processing are shown in Table 5.

## 6. Future Prospect

### 6.1. Smart Sensors and Applications

**Figure 27:**
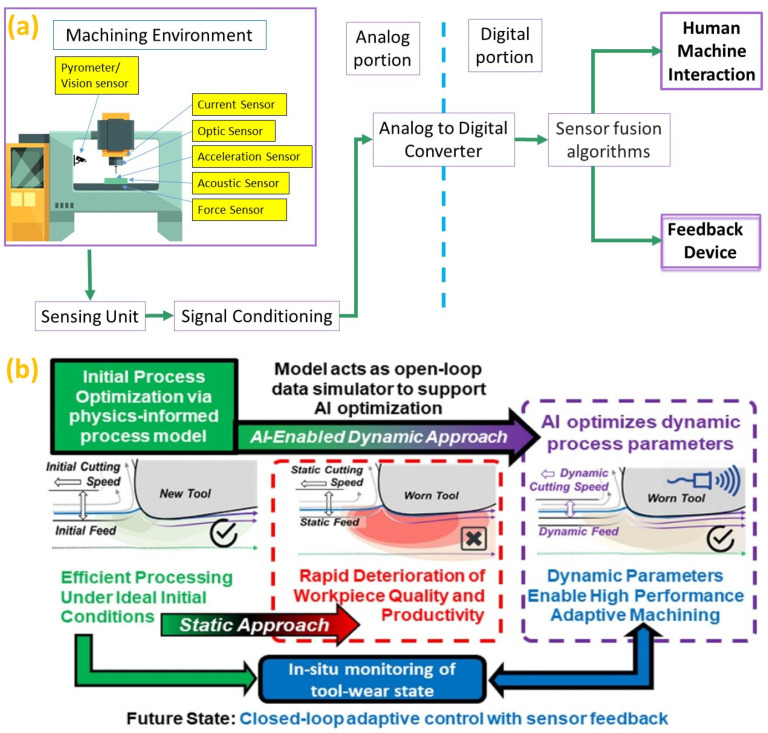
(**a**) Intelligent sensor-based machining condition sensing; (**b**) schematic of AI-enabled dynamic process parameter optimization framework considering progressive tool-wear (reprinted with permission from [232]. Copyright 2021 Society of Manufacturing Engineers (SME)).

Due to the increasing application of I4.0, intelligent sensors have become the driving force of the industrial environment in the digital transformation of the manufacturing process to enhance security, reliability, competitiveness, transparency, and flexibility [228]. These smart sensors are integrated into the subsystems and components of the additive or subtractive machines, which allow for the predictive maintenance, in-situ condition monitoring and performance monitoring during their operation [229]. Usually, subtractive machining has been integrated with conventional sensors. However, some key differences exist between conventional and intelligent sensors, which are created as IoT components to convert real-time situations into digital information to monitor and predict real-time scenarios and take corrective actions instantly [230]. Intelligent sensors’ major tasks include complicated multi-layered activities, such as adjusting sensitivity, collecting raw data, analyzing, filtering and communication [231]. Mainly, the sensor fusion algorithm integrates sensory input, which will appropriately synthesize and help to decrease machine perception uncertainty. The intelligent subtractive system with smart sensor integration is displayed in Figure 27a. Moreover, machine learning has been utilized in static process parameter optimization by maintaining a single set of ‘feed and speed’ throughout a machining operation. However, the process parameters need to be adjusted based on the actual state of the tool/workpiece, and thus, fusion of AI with subtractive manufacturing will be able to control processing parameters well. Therefore, the dynamic tool condition monitoring in a closed-loop feedback system, especially in-situ monitoring of both tool and work piece condition, will be the future state of closed-loop control with sensor feedback in subtractive manufacturing, as illustrated in Figure 27b [232], where in-situ tool-wear measurement will support the real-time adaptive control.

### 6.2. Intelligent 4D Printing

**Figure 28:**
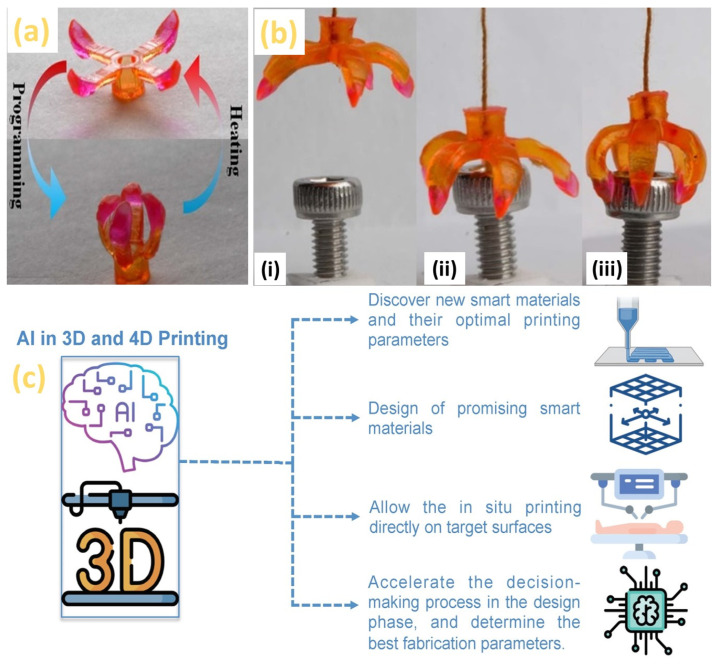
Four-dimensional printing material demonstration (**a**) of the transition between the gripper shape with programming and heating environment [235]; (**b**) thermo-responsive and time-lapsed images of a gripper grabbing an object [235]; and (**c**) possible uses of AI in 3D- and 4D printing applications [237].

Four-dimensional printing, originated from the 3D printing technology, of material added with time results in dynamic characteristics where materials/products reshape with environmental factors (heat, moisture, pH, light, electric energy, magnetic energy) according to a pre-programmed command [233]. Most of the 4D printing research focused on the shape changing ability (such as bending, elongation, twisting) of 4D printed parts [234]. Figure 28a shows the tailorable material (shape memory polymers) used in a 4D printed gripper [235] and Figure 28b shows the object grabbing images of the gripper [235]. The stimulus-responsive materials, interaction mechanisms and mathematical modelling will be required in 4D printing for the prediction of the shape-shifting as a function of time [236]. Moreover, bio-medical applications, such as patient-specific organs fabricated by 3D/4D printing are typically manufactured ex-situ and then transferred to the human body with limited “real-time knowledge” of the target geometry renders mismatch between the printed part and target surfaces [237]. This problem could be addressed by using an ML algorithm to predict the most likely behavior of a phenomenon of 3D- and 4D printing [238]. However, obtaining a large dataset for training the ML algorithm is challenging for prospective organ systems [239]. Therefore, intelligent 3D- and 4D printing [240] are expected to perform the necessary step for the development of personalized anatomical models, as shown in Figure 28c [237].

### 6.3. Machine to Machine (M2M) Communication and Machine to Human (M2H) Interaction

**Figure 29:**
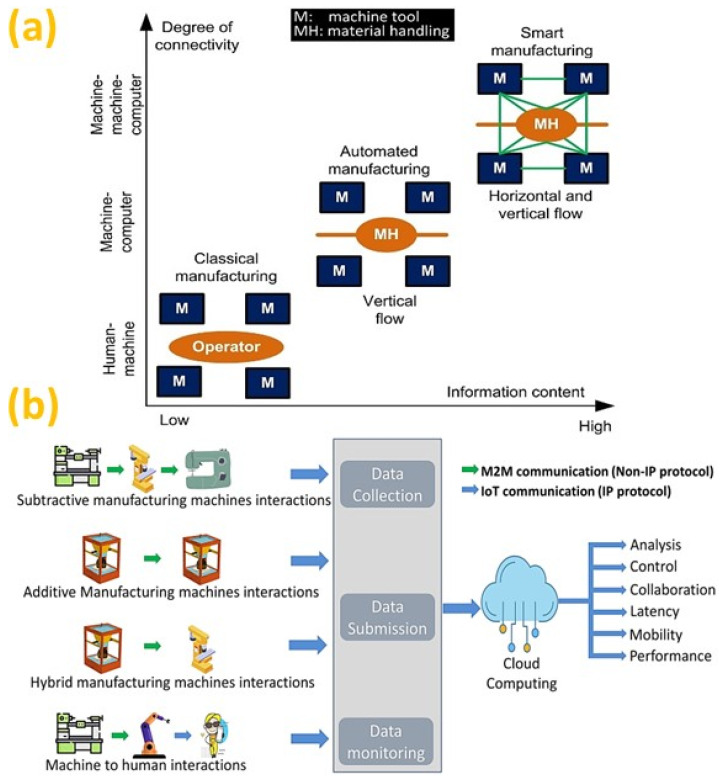
Schematic representation of (**a**) the evolution of connectivity in manufacturing (reprinted with permission from [241]. Copyright 2019 Elsevier B.V.); (**b**) M2M and IoT communication in manufacturing process.

The manufacturing process is becoming automated, as machines can execute repetitive work consistently and efficiently over extended periods without a halt. Moreover, smart manufacturing is emerging from Industry 4.0 by incorporating automated fabrication processes with machine intelligence, instantaneous data monitoring, controlling and optimization [241]. Therefore, information connectivity has evolved from operators as the information carriers to manufacturing equipment connected to computers and networked equipment and computers, as illustrated in Figure 29a [241]. Therefore, the digital manufacturing has progressed significantly due to the advancement of the machine-to-machine (M2M) communication [242] and machine-to-human (M2H) interaction over the cloud platform, making the process autonomous and data driven over the internet [243]. Moreover, I4.0 technologies have transformed both the AM and SM processes in terms of real-time operation management, data collection and analysis [244]. Hence, IoT, CPS and cloud manufacturing will be the enabler for connecting the machines to ensure collaboration both in AM and SM, as illustrated in Figure 29b. The use of block chain (BC) technology could be useful to establish secure M2M communication [245] or efficient device-to-device (D2D) data exchange [246] in the near future.

### 6.4. Cyber Physical System (CPS) and Digital Twin (DT) Driven Manufacturing

**Figure 30:**
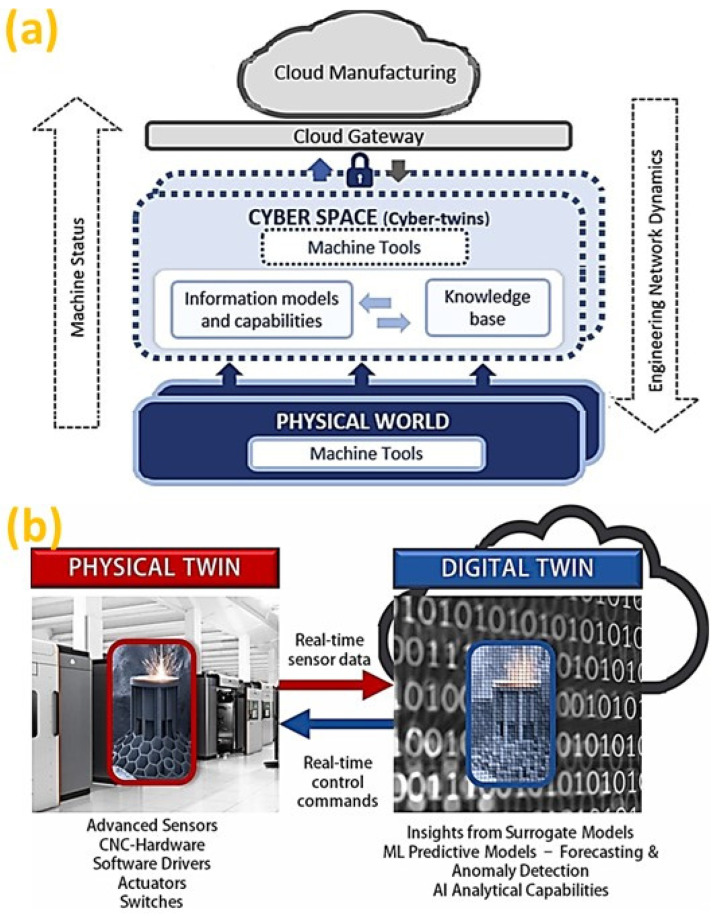
(**a**) Conceptual framework for a CPMT powered cloud manufacturing environment (reprinted with permission from [5]. Copyright 2019 Elsevier B.V.). (**b**) DT process in a real-time diagnostic control capacity (reprinted with permission from [257]. Copyright 2021 Elsevier B.V.).

As the digital transformation through cloud services and resource virtualization allows for intelligent decision making [247], cloud manufacturing is evolving as an integrated cyber-physical system (CPS) to respond quickly and effectively to the changing environment [248]. Therefore, CPS becomes an enabler for ongoing paradigm shifts in manufacturing with interconnected physical and virtualized resources, as well as intelligent search capabilities for design and manufacturing solutions [249]. It is possible to develop a virtual machine tool [250] to act as a building block for “digital-twins” to enable the cyber-physical manufacturing while capturing machining data through sensors [251]. However, since most of the machine tools follow only the toolpath data generated beforehand [252], therefore, it is required for the provision of a smart machine tool for optimal decision support analytics through twinning [253] to support cloud manufacturing, as illustrated in Figure 30a [5]. Thus, a cyber physical machine tool (CPMT) will have a digital space with computing and networking capabilities [254] for the real-time status monitoring of machining processes and controlling the machine tool with built-in computation and intelligence for the decision-making support [255,256].

Nonetheless, DT driven AM is still in its nascency, but it has shown immense potential to transform the additive industry with its autonomous capabilities stemming from the AI embedded in the DT, as illustrated in Figure 30b [257]. Currently, considerable time and cost are being devoted to AM for offline metrology, defect identification and process optimization. DT can mitigate AM defects [258], enhance AM process repeatability [259] and assure AM part quality [123] through real-time closed-loop feedback control. Thus, a DT system looks to reliably address the shortcomings of AM through computational intelligence and real-time collaborative data management [260]. Key application domains include anomaly detection, online condition monitoring, process control, intelligent post-processing and process optimization. This highlights the capacity of DT to widen the future AM application space by improving its robustness and efficiency.

### 6.5. Embracing the Digitalization and Intelligence

**Figure 31:**
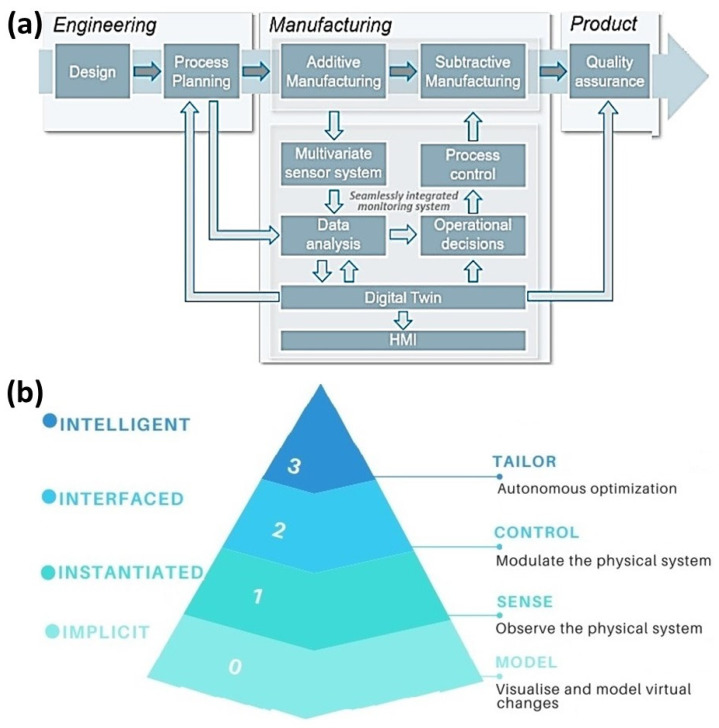
(**a**) framework of the integrated manufacturing monitoring (reprinted with permission from [78]. Copyright 2020 Elsevier B.V.); (**b**) hierarchy of the AM digital twin (reprinted with permission from [267]. Copyright 2022 Elsevier B.V.).

As the intelligent technologies are gradually replacing traditional processes, smart systems are becoming prominent in production-integrated manufacturing through sensor connectivity, communicating technology, cloud computing, simulation, and data-driven modeling [261]. Therefore, it is required to consider the human factors in the current CPS to leverage human intelligence for supporting reliable man-machine interaction to modify the design in real-time and execution of the workflow [262]. Moreover, during a manufacturing operation, human–machine-interface (HMI) can provide near real-time 3D interactive visualization by accessing data from multiple sources and providing information for decision-making from the digital twin [263]. As illustrated in Figure 31a, manufacturing process history is stored in a digital twin where the process chain is divided into three major phases (engineering, manufacturing and end product) [78]. The system intelligence can decide the type and extent of the additive/subtractive process based on the geometry and the metallurgical data in DT. Future prospect remains on predictive analytics by establishing a bidirectional connection between the DT and processes [264], thereby unlocking the M2M and H2M capabilities through the HMI [265]. In this respect, intelligent decision-making through augmented reality (AR) [266] can be a key enabling technology for intelligent human–machine interaction for controlling and monitoring the additive and subtractive manufacturing process.

Moreover, the hierarchy of intelligent DT for manufacturing systems, especially for AM, is illustrated in Figure 31b [267], where the four levels are implicit (based on the knowledge of the system), instantiated (based on the sensor data to enable predictions), interfaced (control and optimization of prediction) and intelligent (enabling complex decision making in a real-time scenario). The use of AI to reason about the current system state and autonomously tailor and optimize parameters in the physical system by adding machine intelligence [268] will be focusing on intelligent and digital manufacturing.

## 7. Conclusions

Intelligent manufacturing has become a new research trend in recent years, as the application of intelligence for monitoring, control and optimization of operations has become typical for both subtractive and additive manufacturing. In subtractive manufacturing (SM), the intelligence is used for real-time sensing, monitoring and control of machining process parameters, i.e., cutting speed, tool temperature, tool wear or tool condition. In additive manufacturing (AM), the machine intelligence is used for autonomous path planning, in-situ monitoring, defect detection and control of printing parameters and sensing and controlling of melt pools. Further automation and intelligence can be achieved by digital and cloud manufacturing by establishing machine-to-machine (M2M) connections, introducing digital twins and applying machine learning and artificial intelligence for process optimization. This article aims to offer a comprehensive review of the state-of-the-art intelligent subtractive and additive manufacturing processes. Despite the wide-ranging research in this field, there has been a shortage of extensive reviews that summarize the impact of intelligence in manufacturing processes. This article aims to fill that gap by providing a thorough overview of the latest developments in this area, highlighting the challenges and perspectives that remain to be addressed. The following key conclusions on the current status and future directions can be drawn from this review:

### 7.1. Current Status

- The integration of artificial intelligence (AI) into machines and software are widely recognized as a key enabler of process automation. However, for AI to fully realize its potential to improve subtractive and additive manufacturing technologies, it is critical that significant advances should be made in the areas of connectivity, sensing, data collection and transmission;
- An on-machine measurement system is imperative for the implementation of automatic compensation during tool path generation, which can be achieved through the implementation of feedback control of the process parameters in intelligent machining. Moreover, the integration of smart technology is crucial for the in-situ evaluation of defects and quality control of additive manufactured (AM) parts;
- The generalization capability of most machine learning (ML) algorithms is well-known. However, it is also widely acknowledged that the variability in the manufacturing process can undermine the effectiveness of these algorithms. Moreover, AI-based predictions are regarded as black-box style indications where the end user has limited access on the decision-making rationale of the model. The lack of clarity due to the complex computing architecture has reduced the trustworthiness of AI predictions, especially when the process chains are dependent on inputs from various operations;
- The post-processing method of additively manufactured parts provides opportunities for improvement in surface quality though the method is deeply integrated with the application and the primary processing techniques. Moreover, conventional finishing methods may prove inadequate in complex structures. To address this challenge, a hybrid process that considers the thermal history of the part is necessary to create a digital thread in the tool path planning for intermediate and final machining operations. Furthermore, it is difficult to capture the intrinsic structure-process–property performance relationship through intelligent techniques. Thus, most of the current intelligent predictive models are material-dependent and may not work for a different material;
- Hybrid or convergent manufacturing is the next-generation manufacturing process, offering infinite possibilities of creating complex-shaped parts with desired dimensional accuracy and surface finish, that are otherwise difficult to obtain by either subtractive or additive manufacturing. However, the current commercial hybrid machines are expensive, and the amortization period is long due to limited use cases. Integration of intelligent and smart manufacturing tools, i.e., in-situ monitoring, sensing, and control and application of ML and AI will be the near future direction of hybrid manufacturing research;
- Digitalization of traditional manufacturing equipment and processes are the path to keep up with the trend of automation and Industry 4.0. Small and medium size industries can be benefit from the digital manufacturing technologies, which will allow control of all the equipment in a factory by monitoring and controlling them from a web cloud. However, there are challenges associated with the implication of digital and smart manufacturing tools. A large number of sensors need to be installed on the machine tool for data collection and storage, which may not be cost-effective for small to medium-scale manufacturing industries. In addition to data collection, the accurate data analysis is challenging;
- The advancement in 3D printing technology has opened new avenues in the field of bioprinting, with 4D printing being at the forefront of this revolution. The ability to create personalized bio-printing solutions through 4D printing applications has generated significant interest in recent times.

### 7.2. Future Prospects

- To overcome the challenges mentioned above and fully realize the potential of intelligent subtractive and additive manufacturing, continued research is required in these areas, including advances in the collection and analysis of process data, and the development of sophisticated and platform independent M2M communication systems that can effectively transmit data in real-time. Moreover, to address limited availability of training data for AI and ML models, future research should focus on revealing the physics of the process and then use the physics-based findings to train the model using ML techniques so that manufacturing science can be revealed with the change of new operating conditions or input data;
- The integration of various smart sensors into real-time monitoring and feedback control systems will offer an in-depth understanding of the hybrid machining process. This allows for a more complete evaluation of the process by dynamically incorporating thermal, acoustic and electromagnetic signals or even other information that was ignored from a machining perspective. Therefore, a complex and innovative algorithm will be needed to process such a data matrix that will ultimately enable the implementation of high-performance adaptive and hybrid manufacturing techniques beyond current perception;
- Future manufacturing trends will not only focus on combining additive and subtractive manufacturing in a single platform but also will incorporate smart sensing technology into the convergent manufacturing system to create an intelligent smart convergent manufacturing system. In comparison to other manufacturing processes, the hybrid systems needed to fabricate complete parts are still low. Thus, the rapid expansion of hybrid processes is needed with adaptive learning, digital metrology, in situ monitoring, and 3D model synchronization for smart factory applications;
- With rapidly evolved cloud manufacturing into an integrated cyber-physical system (CPS) leveraging cloud services, virtualized resources and intelligent decision-making capabilities enable the development of virtual machine tool, which can act as building block for digital twin to facilitate cyber-physical manufacturing. However, to be truly effective, the cloud manufacturing requires the provision of smart machine tools with built-in computation and intelligence to support the optimal decision-making through real-time monitoring of processes. Hence, a cyber-physical machine tool (CPMT) will have a digital space with computing and networking capabilities to provide real-time monitoring and feedback control, for example: edge computing;
- Four-dimensional printing requires a further leap forward in terms of technological sophistication. However, in biomedical applications, such as personalized organ printing, there is a mismatch between the printed part and the target surface due to limited real-time knowledge of the target geometry. To address this challenge, AI-based intelligent 3D and 4D printing can be used to predict the most likely behavior of the printing process and help to develop personalized anatomical models. However, collecting a large enough dataset for training the AI algorithms remains a critical challenge in this field;
- Managing an end-to-end hybrid process requires experienced engineers, designers and operators who are not always available. This creates bottlenecks in scaling up these processes for industrial applications. The future research, thus, will be focused on reducing the barriers for expert users through software and process automation algorithms. In this respect, more emphasis will be needed on digital twin (DT) technology which has created a lot of significant advances in AM and SM processes for evaluating the virtual representation. However, both spaces differ from each other. Therefore, the DT to overcome the divergence for the seamless inclusion of a product’s mechanical and microstructural behavior to precisely obtain physical attributes before the production process;
- Over the past decade, ML algorithms have been widely studied and adopted in various manufacturing-related fields. However, the variability in manufacturing processes can limit the effectiveness of these ML algorithms. To address this challenge, transfer learning (TL) has emerged as a solution, allowing for transferring knowledge acquired from one process variation to another. Despite current limitations in transmission speed and data storage sizes, advancements in AI tools, such as ChatGPT by OpenAI and Bard by Google offer a glimpse into a future where cross-disciplinary topics can be understood in a more integrated manner.

## Figures and Tables

**Table 1:** The advantages and disadvantages of the subtractive, additive and hybrid manufacturing processes.

Process	Advantage	Disadvantage
Subtractive Manufacturing (SM)	Used for wide variety of materials [38]; Achieves excellent dimensional control with tight tolerances and good surface finish [39]; Processes are faster as compared to additive manufacturing in terms of mass production [40]; On-machine-measurement with high accuracy is steadily available, for both probe and beam type, to improve both surface quality and machining process [41].	Material wastage resulting from excessive scrap formation [42]; Not profitable for smaller production runs, as setup and tooling costs are high [43]; Extremely complex shapes cannot be realized [39].
Additive Manufacturing (AM)	Facilitates production of complex near net shaped parts by offering high degree of design freedom [44]; Mass customization is possible by easily altering the product design [40]; Effective utilization of raw material leads to minimal scrap formation and less energy consumption [45]; Dominates over subtractive method in terms of environmental friendliness [46]; Appropriate for small batch production of parts/components [47]; Material density and weight of the part can be controlled effectively through optimal design strategies [48]; On-machine-measurement is possible with both geometrical and thermal approaches [49,50]; Robot-based AM processes are easy to resume, especially with real-time dimensional feedback [51].	AM parts seldom fit the industrial requirements due to the poor surface integrity and dimensional errors [52]; Post-processing operations are inevitable for AM parts/components to enhance their acceptability in industrial applications [53]; Usually not suitable for large sized products owing to smaller build volume [54]; Not applicable to all types of materials [55]; Limitation arises due to the anisotropy in microstructure and mechanical properties [56]; Building strategy in discrete consecutive steps make the process slow and inappropriate for bulk production [57]; Not economical unless the requirement becomes the batch production of extremely complex components [58]; Even with recent developments [59], it is difficult to obtain online measurements for PBF technologies and thus real-time repair.
Hybrid Manufacturing (HM)	Hybridization opens infinite possibilities for enhancing part quality improvement and production rates [60]; Limitations of individual methods can be overcome through hybrid approach [61]. For instance, the combination of AM with SM (CNC milling) facilitates both 3D printing and finishing of the component in a single setup, thereby minimizing the setup error as the printed component does not require shifting to a separate machine [62]; Lowers the production time and minimizes the production cost without compromising on the part quality. In addition, the hybrid processes ensure timely delivery of parts/components and reduction of inventory [63]; Various online measurement approaches are available to facilitate the HM. [64,65].	HM processes demand huge investment and setup costs for the equipment [66]; Challenges arise in scaling the HM setup to fulfil the requirements of mass production as the optimization of the process parameters and effective process control are difficult in a hybrid system [28]; The complexity associated with the setup restricts the large-scale implementation of HM in industries [67]; The switching between AM and SM in a hybrid co-ordinate system can be challenging for CAM designers [68]; Unexpected error or tool damage can arise from the misinterpretation of the AM process, without adequate online feedback [69].

**Table 2:** Classification of additive manufacturing process.

Sl	Process	Material Supply Phase	Example	Phase Change Type	Description
1.	Vat photopolymerization	Liquid	Stereolithography (SLA), Direct Light Processing (DLP), Solid Ground Curing (SGC), Continuous Liquid Interface Production (CLIP), Continuous Direct Light Processing (CDLP)	Photopolymerization	Light-activated polymerization selectively cures liquid photopolymer in a vat.
2.	Material jetting	Liquid	Ink-Jet Printing, PolyJet, Nano Particle Jetting (NPJ), Drop on Demand (DOD)	Photopolymerization	Built material droplets are selectively deposited onto the build platform to solidify and build the model.
3.	Binder jetting or PolyJet	Powder and liquid	Binder Jetting Three-Dimensional Printing (Bj3DP)	Densification	A thin layer of powder materials is selectively applied using a liquid bonding agent.
4.	Powder bed fusion	Powder	Selective Laser Sintering (SLS), Selective Laser Melting (SLM), Electron Beam Powder Bed Fusion (E-PBF), Direct Metal Laser Sintering (DMLS), High-Speed Sintering (HSS), Selective Heat Sintering (SHS)	Sintering or melting	Thermal energy, such as a laser or electron beam, selectively fuses powder material regions.
5.	Directed energy deposition	Powder or wire	Laser Engineered Net Shaping (LENS), Electron Beam Additive Manufacturing (EBAM), Wire Arc Additive Manufacturing (WAAM), Aerosol Jetting (AJ), Directed Light Fabrication (DLF), Laser Deposition Welding (LDW)	Melting	Focused thermal energy is applied to melt and fuse materials simultaneously, as they are deposited on a surface by a nozzle.
6.	Material extrusion	Filament wire	Fused Deposition Modeling (FDM), Fused Pellet Modeling (FPM), Powder Melt Extrusion (PME)	Solidification by cooling	A moving heated extruder head selectively dispenses continuous filament material, which is subsequently deposited via a nozzle or orifice.
7.	Laminated object	Solid	Ultrasonic Consolidation (UC), Ultrasonic Additive Manufacturing (UAM)	No phase change	Heat and pressure are applied to fuse or laminate adhesive-coated sheets of material together to make an item.

**Table 3:** Overview of the sensing techniques used for in-situ monitoring of PBF [123].

Principle of Sensing	Type of Defect	Notes	Ref.
Ultrasonic Testing	Porosity, balling	For qualitative purposes	[124,125,126]
Acoustic Emission Spectroscopy	Overheating, cracking	For qualitative purposes	[127,128,129,130]
Optical Imaging	Powder bed irregularities, overheating	Potential to detect thermal anomalies	[131,132]
Optical Emission Spectroscopy	Overheating, monitor/predict defects	Mostly used in plasma-based processes	[133,134]
Optical Tomography	Balling	For sub-surface detection	[135,136]
X-ray Tomography	Surface roughness, dimensional accuracy	Early phase of development	[137,138]
Optical Coherence Tomography	Powder bed irregularities, lack of fusion defects, keyhole fluctuation, melt pool fluctuation, keyhole pore formation	Limited to surface defects	[139]
Pyrometry	Overheating	Suitable for multiple scan areas	[140,141]
Infrared Imaging	Overheating	Potential to scan entire build area	[141]
In-Situ X-ray Imaging/Diffraction	Keyhole pore formation, melt pool size/shape, powder ejection solidification, phase transformation	For quantitative structural information	[142]

**Table 4:** List of hybrid manufacturing systems/machines.

	Model Name	Configuration
Optomec [172]	LENS 860 Hybrid Open Atmosphere System, LENS 860 Hybrid Controlled Atmosphere	Combines LENS and CNC machining (up to 5 axes)
DMG MORI [173]	LASERTEC 65, 125, 300, 6000	Combines LENS and CNC machining (up to 5 axes)
MAZAK [174]	INTEGREX i-250S AM, INTEGREX i-400 AM, INTEGREX i-600/5X	i-250S AM combines LENS (multiple laser beams) and CNC machining (up to 5 axis), i-400 AM combines LENS (single laser beam) and CNC machining (up to 5 axes), i-600/5X combines wire-arc and CNC machining (up to 5 axes)
Hermle [175]	N/A	Combines proprietary metal-powder-application (MPA) and CNC machining (up to 5 axes)
Fabrisonic [176]	N/A	Combines ultrasonic additive manufacturing and 3 axes CNC machining
3D Metal Forge [177]	H-WAAM	Uses two robotic arms—one for wire-arc additive manufacturing and the other for robotic machining
Hybrid Manufacturing Technologies [178]	AMBIT ONE, AMBIT FLEX, AMBIT EDDY, AMBIT XTRUDE, AMBIT MULTI, AMBIT WAVE, AMBIT SCAN	Develops end effectors for DED (laser), scanning and sensing which attach to CNC machines
3D-Hybrid [179]	Laser, Arc, Cold Spray	Develops end effectors for laser DED, wire-arc DED, and cold-spray.

**Table 5:** Challenges in intelligent post-processing and future directions.

Challenges	Description of the Challenges	Next Step
Expandability	Intelligent techniques are mostly AI-based, where AI models, such as CNNs have been widely employed. However, lack of transparency due to the complex computing architecture has resulted in reduced trustworthiness of AI predictions.	Development of in-situ sensors and sensor fusion in benchmarking training data set.
Lack of data	For several complex processes, such as AM, the generation of a large dataset is very challenging due to the cost and time restrictions.	The training data set representing real-world situations.
Variability in processing requirements	Different technologies demand different post-processing techniques which are extremely difficult to capture through intelligent techniques.	Knowledge of domain expert with AI knowledge is required.
Lack of robustness	The intelligent post-process techniques are often developed for a specific application (mostly DED), machine and controlled test conditions.	CSAM and powder bed fusion AM process need more R&D.
Material dependency	It is difficult to capture the intrinsic process–property–performance relationship through intelligent techniques.	Process fingerprints are introduced.
Integration quality control	Seamless integration of post-processing and final quality compliance in terms of dimensional accuracy, form tolerance and material properties are difficult.	The autonomous models are yet to be developed.
Environment control	Management of coolant for machining vs. inert gas environment for AM can be challenging.	Dry/cryogenic machining with active chip removal.
Other post processing	With near net shape AM parts, polishing and grinding operations may suffice and replace machining.	Improvement for net-shape.

## Data Availability

Not applicable.

## References

[1] Arinez J.F., Chang Q., Gao R.X., Xu C., Zhang J. (2020). Artificial Intelligence in Advanced Manufacturing: Current Status and Future Outlook. J. Manuf. Sci. Eng..

[2] Monostori L., Kádár B., Bauernhansl T., Kondoh S., Kumara S., Reinhart G., Sauer O., Schuh G., Sihn W., Ueda K. (2016). Cyber-Physical Systems in Manufacturing. CIRP Ann..

[3] Lim M.K., Xiong W., Lei Z. (2020). Theory, Supporting Technology and Application Analysis of Cloud Manufacturing: A Systematic and Comprehensive Literature Review. Ind. Manag. Data Syst..

[4] Volpe G., Mangini A.M., Fanti M.P. An Architecture for Digital Processes in Manufacturing with Blockchain, Docker and Cloud Storage. Proceedings of the 2021 IEEE 17th International Conference on Automation Science and Engineering (CASE).

[5] Liu C., Vengayil H., Lu Y., Xu X. (2019). A Cyber-Physical Machine Tools Platform Using OPC UA and MTConnect. J. Manuf. Syst..

[6] Cho J., Kang S., Kim K. (2022). Real-Time Precise Object Segmentation Using a Pixel-Wise Coarse-Fine Method with Deep Learning for Automated Manufacturing. J. Manuf. Syst..

[7] Boccella A.R., Centobelli P., Cerchione R., Murino T., Riedel R. (2020). Evaluating Centralized and Heterarchical Control of Smart Manufacturing Systems in the Era of Industry 4.0. Appl. Sci..

[8] Wang S., Wan J., Li D., Zhang C. (2016). Implementing Smart Factory of Industrie 4.0: An Outlook. Int. J. Distrib. Sens. Netw..

[9] Zhang Y., Qian C., Lv J., Liu Y. (2017). Agent and Cyber-Physical System Based Self-Organizing and Self-Adaptive Intelligent Shopfloor. IEEE Trans. Ind. Inf..

[10] Sari T., Gules H.K., Yigitol B. (2020). Awareness and Readiness of Industry 4.0: The Case of Turkish Manufacturing Industry. Adv. Prod. Eng. Manag..

[11] Thames L., Schaefer D., Thames L., Schaefer D. (2017). Industry 4.0: An Overview of Key Benefits, Technologies, and Challenges. Cybersecurity for Industry 4.0.

[12] Yuan C., Li G., Kamarthi S., Jin X., Moghaddam M. (2022). Trends in Intelligent Manufacturing Research: A Keyword Co-Occurrence Network Based Review. J. Intell. Manuf..

[13] Sarker I.H. (2022). AI-Based Modeling: Techniques, Applications and Research Issues Towards Automation, Intelligent and Smart Systems. SN Comput. Sci..

[14] Gajsek B., Marolt J., Rupnik B., Lerher T., Sternad M. (2019). Using Maturity Model and Discrete-Event Simulation for Industry 4.0 Implementation. Int. J. Simul. Model..

[15] Caggiano A. (2018). Cloud-Based Manufacturing Process Monitoring for Smart Diagnosis Services. Int. J. Comput. Integr. Manuf..

[16] Cheng K., Niu Z.-C., Wang R.C., Rakowski R., Bateman R. (2017). Smart Cutting Tools and Smart Machining: Development Approaches, and Their Implementation and Application Perspectives. Chin. J. Mech. Eng..

[17] Ong P., Lee W.K., Lau R.J.H. (2019). Tool Condition Monitoring in CNC End Milling Using Wavelet Neural Network Based on Machine Vision. Int. J. Adv. Manuf. Technol..

[18] Liu L., Zhang X., Wan X., Zhou S., Gao Z. (2022). Digital Twin-Driven Surface Roughness Prediction and Process Parameter Adaptive Optimization. Adv. Eng. Inform..

[19] Chuo Y.S., Lee J.W., Mun C.H., Noh I.W., Rezvani S., Kim D.C., Lee J., Lee S.W., Park S.S. (2022). Artificial Intelligence Enabled Smart Machining and Machine Tools. J. Mech. Sci. Technol..

[20] Araújo N., Pacheco V., Costa L. (2021). Smart Additive Manufacturing: The Path to the Digital Value Chain. Technologies.

[21] Brown K.A., Gu G.X. (2021). Dimensions of Smart Additive Manufacturing. Adv. Intell. Syst..

[22] Kim D.B., Witherell P., Lipman R., Feng S.C. (2015). Streamlining the Additive Manufacturing Digital Spectrum: A Systems Approach. Addit. Manuf..

[23] Kunovjanek M., Knofius N., Reiner G. (2022). Additive Manufacturing and Supply Chains—A Systematic Review. Prod. Plan. Control..

[24] Wang C., Tan X.P., Tor S.B., Lim C.S. (2020). Machine Learning in Additive Manufacturing: State-of-the-Art and Perspectives. Addit. Manuf..

[25] Oleff A., Küster B., Stonis M., Overmeyer L. (2021). Process Monitoring for Material Extrusion Additive Manufacturing: A State-of-the-Art Review. Prog. Addit. Manuf..

[26] Du W., Bai Q., Zhang B. (2016). A Novel Method for Additive/Subtractive Hybrid Manufacturing of Metallic Parts. Procedia Manuf..

[27] Bhaduri D., Penchev P., Batal A., Dimov S., Soo S.L., Sten S., Harrysson U., Zhang Z., Dong H. (2017). Laser Polishing of 3D Printed Mesoscale Components. Appl. Surf. Sci..

[28] Boban J., Ahmed A., Jithinraj E.K., Rahman M.A., Rahman M. (2022). Polishing of Additive Manufactured Metallic Components: Retrospect on Existing Methods and Future Prospects. Int. J. Adv. Manuf. Technol..

[29] Manogharan G., Wysk R., Harrysson O., Aman R. (2015). AIMS—A Metal Additive-Hybrid Manufacturing System: System Architecture and Attributes. Procedia Manuf..

[30] Kerbrat O., Mognol P., Hascoët J.-Y. (2011). A New DFM Approach to Combine Machining and Additive Manufacturing. Comput. Ind..

[31] Dilberoglu U.M., Gharehpapagh B., Yaman U., Dolen M. (2017). The Role of Additive Manufacturing in the Era of Industry 4.0. Procedia Manuf..

[32] Pragana J.P.M., Sampaio R.F.V., Bragança I.M.F., Silva C.M.A., Martins P.A.F. (2021). Hybrid Metal Additive Manufacturing: A State–of–the-Art Review. Adv. Ind. Manuf. Eng..

[33] Grzesik W. (2018). Hybrid Additive and Subtractive Manufacturing Processes and Systems: A Review. J. Mach. Eng..

[34] Behandish M., Nelaturi S., de Kleer J. (2018). Automated Process Planning for Hybrid Manufacturing. Comput. -Aided Des..

[35] Abdulhameed O., Al-Ahmari A.M., Ameen W., Mian S.H. (2018). Novel Dynamic CAPP System for Hybrid Additive–Subtractive–Inspection Process. Rapid Prototyp. J..

[36] Dávila J.L., Neto P.I., Noritomi P.Y., Coelho R.T., da Silva J.V.L. (2020). Hybrid Manufacturing: A Review of the Synergy between Directed Energy Deposition and Subtractive Processes. Int. J. Adv. Manuf. Technol..

[37] Jena M.C., Mishra S.K., Moharana H.S. (2020). Application of Industry 4.0 to Enhance Sustainable Manufacturing. Environ. Prog. Sustain. Energy.

[38] Smith C.S., Wright P.K. (1996). CyberCut: A World Wide Web Based Design-to-Fabrication Tool. J. Manuf. Syst..

[39] Chand R., Sharma V.S., Trehan R., Gupta M.K., Sarikaya M. (2022). Investigating the Dimensional Accuracy and Surface Roughness for 3D Printed Parts Using a Multi-Jet Printer. J. Mater. Eng. Perform..

[40] Ngo T.D., Kashani A., Imbalzano G., Nguyen K.T.Q., Hui D. (2018). Additive Manufacturing (3D Printing): A Review of Materials, Methods, Applications and Challenges. Compos. B Eng..

[41] DIng D., Zhao Z., Huang R., Dai C., Zhang X., Xu T., Fu Y. (2021). Error Modeling and Path Planning for Freeform Surfaces by Laser Triangulation On-Machine Measurement. IEEE Trans. Instrum. Meas..

[42] Sathish K., Kumar S.S., Magal R.T., Selvaraj V., Narasimharaj V., Karthikeyan R., Sabarinathan G., Tiwari M., Kassa A.E. (2022). A Comparative Study on Subtractive Manufacturing and Additive Manufacturing. Adv. Mater. Sci. Eng..

[43] Yusuf S.M., Cutler S., Gao N. (2019). Review: The Impact of Metal Additive Manufacturing on the Aerospace Industry. Metals.

[44] Korpela M., Riikonen N., Piili H., Salminen A., Nyrhilä O. (2020). Additive Manufacturing—Past, Present, and the Future. Tech. Econ. Soc. Eff. Manuf. 4.0.

[45] Pereira T., Kennedy J.V., Potgieter J. (2019). A Comparison of Traditional Manufacturing vs Additive Manufacturing, the Best Method for the Job. Procedia Manuf..

[46] Paris H., Mokhtarian H., Coatanéa E., Museau M., Ituarte I.F. (2016). Comparative Environmental Impacts of Additive and Subtractive Manufacturing Technologies. CIRP Ann..

[47] Boban J., Ahmed A. (2022). Electric Discharge Assisted Post-Processing Performance of High Strength-to-Weight Ratio Alloys Fabricated Using Metal Additive Manufacturing. CIRP J. Manuf. Sci. Technol..

[48] Ingarao G., Priarone P.C. (2020). A Comparative Assessment of Energy Demand and Life Cycle Costs for Additive- and Subtractive-Based Manufacturing Approaches. J. Manuf. Process..

[49] Sdvizhenskii P.A., Lednev V.N., Asyutin R.D., Grishin M.Y., Tretyakov R.S., Pershin S.M. (2020). Online Laser-Induced Breakdown Spectroscopy for Metal-Particle Powder Flow Analysis during Additive Manufacturing. J. Anal. At. Spectrom..

[50] Xia C., Pan Z., Polden J., Li H., Xu Y., Chen S., Zhang Y. (2020). A Review on Wire Arc Additive Manufacturing: Monitoring, Control and a Framework of Automated System. J. Manuf. Syst..

[51] Chabot A., Laroche N., Carcreff E., Rauch M., Hascoët J.Y. (2020). Towards Defect Monitoring for Metallic Additive Manufacturing Components Using Phased Array Ultrasonic Testing. J. Intell. Manuf..

[52] Cao L., Li J., Hu J., Liu H., Wu Y., Zhou Q. (2021). Optimization of Surface Roughness and Dimensional Accuracy in LPBF Additive Manufacturing. Opt. Laser Technol..

[53] Boban J., Ahmed A., Rahman M.A., Rahman M. (2020). Wire Electrical Discharge Polishing of Additive Manufactured Metallic Components. Procedia CIRP.

[54] Abdulhameed O., Al-Ahmari A., Ameen W., Mian S.H. (2019). Additive Manufacturing: Challenges, Trends, and Applications. Adv. Mech. Eng..

[55] Bourell D., Kruth J.P., Leu M., Levy G., Rosen D., Beese A.M., Clare A. (2017). Materials for Additive Manufacturing. CIRP Ann..

[56] Kok Y., Tan X.P., Wang P., Nai M.L.S., Loh N.H., Liu E., Tor S.B. (2018). Anisotropy and Heterogeneity of Microstructure and Mechanical Properties in Metal Additive Manufacturing: A Critical Review. Mater. Des..

[57] Arias-González F., Barro O., del Val J., Lusquiños F., Fernández-Arias M., Comesaña R., Riveiro A., Pou J. (2021). Laser-Directed Energy Deposition: Principles and Applications. Addit. Manuf..

[58] Atzeni E., Salmi A. (2012). Economics of Additive Manufacturing for End-Usable Metal Parts. Int. J. Adv. Manuf. Technol..

[59] Yakout M., Phillips I., Elbestawi M.A., Fang Q. (2021). In-Situ Monitoring and Detection of Spatter Agglomeration and Delamination during Laser-Based Powder Bed Fusion of Invar 36. Opt. Laser Technol..

[60] Lalegani Dezaki M., Serjouei A., Zolfagharian A., Fotouhi M., Moradi M., Ariffin M.K.A., Bodaghi M. (2022). A Review on Additive/Subtractive Hybrid Manufacturing of Directed Energy Deposition (DED) Process. Adv. Powder Mater..

[61] Newman S.T., Zhu Z., Dhokia V., Shokrani A. (2015). Process Planning for Additive and Subtractive Manufacturing Technologies. CIRP Ann..

[62] Liu C., Yan D., Tan J., Mai Z., Cai Z., Dai Y., Jiang M., Wang P., Liu Z., Li C.C. (2020). Development and Experimental Validation of a Hybrid Selective Laser Melting and CNC Milling System. Addit. Manuf..

[63] Chen N., Frank M. (2019). Process Planning for Hybrid Additive and Subtractive Manufacturing to Integrate Machining and Directed Energy Deposition. Procedia Manuf..

[64] Xu K., Li Y., Liu C., Liu X., Hao X., Gao J., Maropoulos P.G. (2020). Advanced Data Collection and Analysis in Data-Driven Manufacturing Process. Chin. J. Mech. Eng..

[65] Amanullah A.N.M., Murshiduzzaman, Saleh T., Khan R. (2017). Design and Development of a Hybrid Machine Combining Rapid Prototyping and CNC Milling Operation. Procedia Eng..

[66] Boban J., Ahmed A. (2021). Improving the Surface Integrity and Mechanical Properties of Additive Manufactured Stainless Steel Components by Wire Electrical Discharge Polishing. J. Mater. Process. Technol..

[67] Krakhmalev P., Sebbe N.P.V., Fernandes F., Sousa V.F.C., Silva F.J.G. (2022). Hybrid Manufacturing Processes Used in the Production of Complex Parts: A Comprehensive Review. Metals.

[68] Li L., Haghighi A., Yang Y. (2018). A Novel 6-Axis Hybrid Additive-Subtractive Manufacturing Process: Design and Case Studies. J. Manuf. Process..

[69] Cortina M., Arrizubieta J., Ruiz J., Ukar E., Lamikiz A. (2018). Latest Developments in Industrial Hybrid Machine Tools That Combine Additive and Subtractive Operations. Materials.

[70] Wang L. (2019). From intelligence science to intelligent manufacturing. Engineering.

[71] Iqbal A., Zhao G., Suhaimi H., He N., Hussain G., Zhao W. (2020). Readiness of Subtractive and Additive Manufacturing and Their Sustainable Amalgamation from the Perspective of Industry 4.0: A Comprehensive Review. Int. J. Adv. Manuf. Technol..

[72] Aggour K.S., Gupta V.K., Ruscitto D., Ajdelsztajn L., Bian X., Brosnan K.H., Chennimalai Kumar N., Dheeradhada V., Hanlon T., Iyer N. (2019). Artificial Intelligence/Machine Learning in Manufacturing and Inspection: A GE Perspective. MRS Bull..

[73] Caggiano A., Zhang J., Alfieri V., Caiazzo F., Gao R., Teti R. (2019). Machine Learning-Based Image Processing for on-Line Defect Recognition in Additive Manufacturing. CIRP Ann..

[74] Poole D.L., Mackworth A.K., Goebel R. (1998). Computational Intelligence: A Logical Approach.

[75] Kim D.-H., Song J.-Y. (2006). Knowledge-Evolutionary Intelligent Machine-Tools —Part 1: Design of Dialogue Agent Based on Standard Platform. J. Mech. Sci. Technol..

[76] Kim D.-H., Song J.-Y., Lee J.-H., Cha S.-K. (2009). Development and Evaluation of Intelligent Machine Tools Based on Knowledge Evolution in M2M Environment. J. Mech. Sci. Technol..

[77] Lee S.W., Lee H.K. (2009). Rule-Based Cutting Condition Recommendation System for Intelligent Machine Tools. J. Mech. Sci. Technol..

[78] Reisch R., Hauser T., Kamps T., Knoll A. (2020). Robot Based Wire Arc Additive Manufacturing System with Context-Sensitive Multivariate Monitoring Framework. Procedia Manuf..

[79] Verl A., Lechler A., Schlechtendahl J. (2012). Glocalized Cyber Physical Production Systems. Prod. Eng. Res. Devel..

[80] Stentoft J., Olhager J., Heikkilä J., Thoms L. (2016). Manufacturing Backshoring: A Systematic Literature Review. Oper. Manag. Res..

[81] Tranfield D., Denyer D., Smart P. (2003). Towards a Methodology for Developing Evidence-Informed Management Knowledge by Means of Systematic Review. Br. J. Manag..

[82] Akhras G. (2000). Smart materials and smart systems for the future. Can. Mil. J..

[83] Ricquebourg V., Menga D., Durand D., Marhic B., Delahoche L., Loge C. The Smart Home Concept: Our Immediate Future. Proceedings of the 2006 1ST IEEE International Conference on E-Learning in Industrial Electronics.

[84] Kusiak A. (2018). Smart Manufacturing. Int. J. Prod. Res..

[85] Deshayes L., Welsch L., Donmez A., Ivester R., Gilsinn D., Rhorer R., Whitenton E., Potra F., Brissaud D., Tichkiewitch S., Zwolinski P. (2006). Smart Machining Systems: Issues and Research Trends. Innovation in Life Cycle Engineering and Sustainable Development.

[86] Imad M., Hosseini A., Kishawy H.A. (2019). Optimization Methodologies in Intelligent Machining Systems—A Review. IFAC-PapersOnLine.

[87] Chen M., Wang C., An Q., Ming W. (2018). Tool Path Strategy and Cutting Process Monitoring in Intelligent Machining. Front. Mech. Eng..

[88] Zhang J., Starly B., Cai Y., Cohen P.H., Lee Y.-S. (2017). Particle Learning in Online Tool Wear Diagnosis and Prognosis. J. Manuf. Process..

[89] Wang J., Li Y., Zhao R., Gao R.X. (2020). Physics Guided Neural Network for Machining Tool Wear Prediction. J. Manuf. Syst..

[90] Li Z., Liu R., Wu D. (2019). Data-Driven Smart Manufacturing: Tool Wear Monitoring with Audio Signals and Machine Learning. J. Manuf. Process..

[91] Gomes M.C., Brito L.C., da Silva M., Viana Duarte M.A. (2021). Tool Wear Monitoring in Micromilling Using Support Vector Machine with Vibration and Sound Sensors. Precis. Eng..

[92] Li Y., Liu X., Gao J.X., Maropoulos P.G. (2012). A Dynamic Feature Information Model for Integrated Manufacturing Planning and Optimization. CIRP Ann..

[93] Liu X., Li Y., Gao J. (2016). A Multi-Perspective Dynamic Feature Concept in Adaptive NC Machining of Complex Freeform Surfaces. Int. J. Adv. Manuf. Technol..

[94] Li Y., Liu C., Gao J.X., Shen W. (2015). An Integrated Feature-Based Dynamic Control System for on-Line Machining, Inspection and Monitoring. Integr. Comput. Aided Eng..

[95] Zhao X., Zheng L., Wang Y., Zhang Y. (2022). Services-Oriented Intelligent Milling for Thin-Walled Parts Based on Time-Varying Information Model of Machining System. Int. J. Mech. Sci..

[96] Cheung C.F., Kong L.B., Lee W.B., To S. (2006). Modelling and Simulation of Freeform Surface Generation in Ultra-Precision Raster Milling. Proc. Inst. Mech. Eng. Part B J. Eng. Manuf..

[97] Chen Y.-L., Wang S., Shimizu Y., Ito S., Gao W., Ju B.-F. (2015). An In-Process Measurement Method for Repair of Defective Microstructures by Using a Fast Tool Servo with a Force Sensor. Precis. Eng..

[98] Gao W., Haitjema H., Fang F.Z., Leach R.K., Cheung C.F., Savio E., Linares J.M. (2019). On-Machine and in-Process Surface Metrology for Precision Manufacturing. CIRP Ann..

[99] Noor W.I., Saleh T., Rashid M.A.N., Mohd Ibrahim A., Ali M.S.M. (2021). Dual-Stage Artificial Neural Network (ANN) Model for Sequential LBMM-ΜEDM-Based Micro-Drilling. Int. J. Adv. Manuf. Technol..

[100] Rahman M.A., Ahmed A., Mia M. (2021). Trends in Electrical Discharge Machining of Ti- and Ni-Based Superalloys: Macro-Micro-Compound Arc/Spark/Melt Process. Micro Electro-Fabrication.

[101] Joshi S.N., Pande S.S. (2011). Intelligent Process Modeling and Optimization of Die-Sinking Electric Discharge Machining. Appl. Soft Comput..

[102] Kao J.Y., Tarng Y.S. (1997). A Neutral-Network Approach for the on-Line Monitoring of the Electrical Discharge Machining Process. J. Mater. Process. Technol..

[103] Ahmed A., Fardin A., Tanjilul M., Wong Y.S., Rahman M., Senthil Kumar A. (2018). A Comparative Study on the Modelling of EDM and Hybrid Electrical Discharge and Arc Machining Considering Latent Heat and Temperature-Dependent Properties of Inconel 718. Int. J. Adv. Manuf. Technol..

[104] Zhang X., Liu Y., Wu X., Niu Z. (2020). Intelligent Pulse Analysis of High-Speed Electrical Discharge Machining Using Different RNNs. J. Intell. Manuf..

[105] Lee C.H., Lai T.S. (2021). An Intelligent System for Improving Electric Discharge Machining Efficiency Using Artificial Neural Network and Adaptive Control of Debris Removal Operations. IEEE Access.

[106] Jarin S., Saleh T. (2019). Artificial Neural Network Modelling and Analysis of Carbon Nanopowder Mixed Micro Wire Electro Discharge Machining of Gold Coated Doped Silicon. Int. J. Mater. Eng. Innov..

[107] Thankachan T., Soorya Prakash K., Malini R., Ramu S., Sundararaj P., Rajandran S., Rammasamy D., Jothi S. (2019). Prediction of Surface Roughness and Material Removal Rate in Wire Electrical Discharge Machining on Aluminum Based Alloys/Composites Using Taguchi Coupled Grey Relational Analysis and Artificial Neural Networks. Appl. Surf. Sci..

[108] Wuest T., Weimer D., Irgens C., Thoben K.D. (2016). Machine Learning in Manufacturing: Advantages, Challenges, and Applications. Prod. Manuf. Res..

[109] Patange A.D., Jegadeeshwaran R. (2021). A Machine Learning Approach for Vibration-Based Multipoint Tool Insert Health Prediction on Vertical Machining Centre (VMC). Measurement.

[110] Zacharia K., Krishnakumar P. (2020). Chatter Prediction in High Speed Machining of Titanium Alloy (Ti-6Al-4V) Using Machine Learning Techniques. Mater. Today Proc..

[111] Oleaga I., Pardo C., Zulaika J.J., Bustillo A. (2018). A Machine-Learning Based Solution for Chatter Prediction in Heavy-Duty Milling Machines. Measurement.

[112] Unver H.O., Sener B. (2022). Exploring the Potential of Transfer Learning for Chatter Detection. Procedia Comput. Sci..

[113] Wang Y., Wang Y., Zheng L., Zhou J. (2022). Online Surface Roughness Prediction for Assembly Interfaces of Vertical Tail Integrating Tool Wear under Variable Cutting Parameters. Sensors.

[114] Standard Terminology for Additive Manufacturing—General Principles—Terminology. https://www.astm.org/f3177-15.html.

[115] Bidare P., Jiménez A., Hassanin H., Essa K. (2022). Porosity, Cracks, and Mechanical Properties of Additively Manufactured Tooling Alloys: A Review. Adv. Manuf..

[116] Cunningham R.W. (2018). Defect Formation Mechanisms in Powder-Bed Metal Additive Manufacturing. Ph.D. Thesis.

[117] Svetlizky D., Das M., Zheng B., Vyatskikh A.L., Bose S., Bandyopadhyay A., Schoenung J.M., Lavernia E.J., Eliaz N. (2021). Directed Energy Deposition (DED) Additive Manufacturing: Physical Characteristics, Defects, Challenges and Applications. Mater. Today.

[118] Bidare P., Bitharas I., Ward R.M., Attallah M.M., Moore A.J. (2018). Laser Powder Bed Fusion in High-Pressure Atmospheres. Int. J. Adv. Manuf. Technol..

[119] Ferrar B., Mullen L., Jones E., Stamp R., Sutcliffe C.J. (2012). Gas Flow Effects on Selective Laser Melting (SLM) Manufacturing Performance. J. Mater. Process. Technol..

[120] Talib Mohammed M. (2018). Mechanical Properties of SLM-Titanium Materials for Biomedical Applications: A Review. Mater. Today Proc..

[121] Bax B., Rajput R., Kellet R., Reisacher M. (2018). Systematic Evaluation of Process Parameter Maps for Laser Cladding and Directed Energy Deposition. Addit. Manuf..

[122] Ng G.K.L., Jarfors A.E.W., Bi G., Zheng H.Y. (2009). Porosity Formation and Gas Bubble Retention in Laser Metal Deposition. Appl. Phys. A.

[123] McCann R., Obeidi M.A., Hughes C., McCarthy É., Egan D.S., Vijayaraghavan R.K., Joshi A.M., Acinas Garzon V., Dowling D.P., McNally P.J. (2021). In-Situ Sensing, Process Monitoring and Machine Control in Laser Powder Bed Fusion: A Review. Addit. Manuf..

[124] Rieder H., Spies M., Bamberg J., Henkel B. (2016). On- and Offline Ultrasonic Characterization of Components Built by SLM Additive Manufacturing. AIP Conference Proceedings.

[125] Rieder H., Dillhöfer A., Spies M., Bamberg J., Hess T. (2015). Ultrasonic Online Monitoring of Additive Manufacturing Processes Based on Selective Laser Melting. AIP Conference Proceedings.

[126] Honarvar F., Varvani-Farahani A. (2020). A Review of Ultrasonic Testing Applications in Additive Manufacturing: Defect Evaluation, Material Characterization, and Process Control. Ultrasonics.

[127] Wasmer K., Le-Quang T., Meylan B., Shevchik S.A. (2019). In Situ Quality Monitoring in AM Using Acoustic Emission: A Reinforcement Learning Approach. J. Mater. Eng. Perform..

[128] Ye D., Hong G.S., Zhang Y., Zhu K., Fuh J.Y.H. (2018). Defect Detection in Selective Laser Melting Technology by Acoustic Signals with Deep Belief Networks. Int. J. Adv. Manuf. Technol..

[129] Shevchik S.A., Kenel C., Leinenbach C., Wasmer K. (2018). Acoustic Emission for in Situ Quality Monitoring in Additive Manufacturing Using Spectral Convolutional Neural Networks. Addit. Manuf..

[130] Smith R.J., Hirsch M., Patel R., Li W., Clare A.T., Sharples S.D. (2016). Spatially Resolved Acoustic Spectroscopy for Selective Laser Melting. J. Mater. Process. Technol..

[131] Yadroitsev I., Krakhmalev P., Yadroitsava I. (2014). Selective Laser Melting of Ti6Al4V Alloy for Biomedical Applications: Temperature Monitoring and Microstructural Evolution. J. Alloy. Compd..

[132] Craeghs T., Clijsters S., Yasa E., Kruth J.-P. (2011). Online Quality Control of Selective Laser Melting. 2011 International Solid Freeform Fabrication Symposium.

[133] Nassar A.R., Spurgeon T.J., Reutzel E.W. (2014). Sensing Defects during Directed-Energy Additive Manufacturing of Metal Parts Using Optical Emissions Spectroscopy. 2014 International Solid Freeform Fabrication Symposium.

[134] Dunbar A.J., Nassar A.R., Reutzel E.W., Blecher J.J. (2015). A real-time communication architecture for metal powder bed fusion additive manufacturing. 2016 International Solid Freeform Fabrication Symposium.

[135] Mohr G., Altenburg S.J., Ulbricht A., Heinrich P., Baum D., Maierhofer C., Hilgenberg K. (2020). In-Situ Defect Detection in Laser Powder Bed Fusion by Using Thermography and Optical Tomography—Comparison to Computed Tomography. Metals.

[136] Zenzinger G., Bamberg J., Ladewig A., Hess T., Henkel B., Satzger W. (2015). Process Monitoring of Additive Manufacturing by Using Optical Tomography. AIP Conference Proceedings.

[137] du Plessis A., Yadroitsev I., Yadroitsava I., le Roux S.G. (2018). X-ray Microcomputed Tomography in Additive Manufacturing: A Review of the Current Technology and Applications. 3D Print Addit. Manuf..

[138] du Plessis A., Yadroitsava I., Yadroitsev I. (2020). Effects of Defects on Mechanical Properties in Metal Additive Manufacturing: A Review Focusing on X-ray Tomography Insights. Mater. Des..

[139] Kanko J.A., Sibley A.P., Fraser J.M. (2016). In Situ Morphology-Based Defect Detection of Selective Laser Melting through Inline Coherent Imaging. J. Mater. Process. Technol..

[140] Mahato V., Obeidi M.A., Brabazon D., Cunningham P. (2020). An Evaluation of Classification Methods for 3D Printing Time-Series Data. IFAC-PapersOnLine.

[141] Berumen S., Bechmann F., Lindner S., Kruth J.-P., Craeghs T. (2010). Quality Control of Laser and Powder Bed-Based Additive Manufacturing (AM) Technologies. Phys. Procedia.

[142] Zhao C., Fezzaa K., Cunningham R.W., Wen H., de Carlo F., Chen L., Rollett A.D., Sun T. (2017). Real-Time Monitoring of Laser Powder Bed Fusion Process Using High-Speed X-ray Imaging and Diffraction. Sci. Rep..

[143] Mani M., Feng S., Brandon L., Donmez A., Moylan S., Fesperman R. (2015). Measurement science needs for real-time control of additive manufacturing powder-bed fusion processes. Additive Manufacturing Handbook.

[144] Vlasea M.L., Lane B., Lopez F., Mekhontsev S., Donmez A. (2015). Development of powder bed fusion additive manufacturing test bed for enhanced real-time process control. 2015 International Solid Freeform Fabrication Symposium.

[145] Kruth J.-P., Mercelis P., Van Vaerenbergh J., Craeghs T. (2007). Feedback Control of Selective Laser Melting.

[146] Razvi S.S., Feng S., Narayanan A., Lee Y.-T.T., Witherell P. (2019). A Review of Machine Learning Applications in Additive Manufacturing.

[147] Du C.-J., Sun D.-W. (2006). Learning Techniques Used in Computer Vision for Food Quality Evaluation: A Review. J. Food Eng..

[148] Meng L., McWilliams B., Jarosinski W., Park H.-Y., Jung Y.-G., Lee J., Zhang J. (2020). Machine Learning in Additive Manufacturing: A Review. JOM.

[149] Yao X., Moon S.K., Bi G. (2017). A Hybrid Machine Learning Approach for Additive Manufacturing Design Feature Recommendation. Rapid Prototyp. J..

[150] Chen D., Wang P., Pan R., Zha C., Fan J., Kong S., Li N., Li J., Zeng Z. (2021). Research on in Situ Monitoring of Selective Laser Melting: A State of the Art Review. Int. J. Adv. Manuf. Technol..

[151] Zhang Y., Yan W. Applications of Machine Learning in Metal Powder-Bed Fusion in-Process Monitoring and Control: Status and Challenges. J. Intell. Manuf..

[152] Johnson N.S., Vulimiri P.S., To A.C., Zhang X., Brice C.A., Kappes B.B., Stebner A.P. (2020). Invited Review: Machine Learning for Materials Developments in Metals Additive Manufacturing. Addit. Manuf..

[153] Zhang B., Liu S., Shin Y.C. (2019). In-Process Monitoring of Porosity during Laser Additive Manufacturing Process. Addit. Manuf..

[154] de la Yedra A., Pfleger M., Aramendi B., Cabeza M., Zubiri F., Mitter T., Reitinger B., Scherleitner E. (2019). Online Cracking Detection by Means of Optical Techniques in Laser-Cladding Process. Struct. Control Health Monit..

[155] Scime L., Beuth J. (2019). Using Machine Learning to Identify In-Situ Melt Pool Signatures Indicative of Flaw Formation in a Laser Powder Bed Fusion Additive Manufacturing Process. Addit. Manuf..

[156] de Oliveira D., Gomes M.C., dos Santos A.G., Ribeiro K.S.B., Vasques I.J., Coelho R.T., da Silva M.B., Hung N.W. (2022). Abrasive and Non-Conventional Post-Processing Techniques to Improve Surface Finish of Additively Manufactured Metals: A Review. Prog. Addit. Manuf..

[157] Sibanda P.S., Carr P., Ryan M., Bigot S. (2019). State of the Art in Surface Finish of Metal Additive Manufactured Parts. Adv. Transdiscipl. Eng..

[158] Petri K.L., Billo R.E., Bidanda B. (1998). A Neural Network Process Model for Abrasive Flow Machining Operations. J. Manuf. Syst..

[159] Markopoulos A., Vaxevanidis N.M., Petropoulos G., Manolakos D.E. (2006). Artificial Neural Networks Modeling of Surface Finish in Electro-Discharge Machining of Tool Steels. Proceedings of the Volume 4: Fatigue and Fracture, Heat Transfer, Internal Combustion Engines, Manufacturing, and Technology and Society.

[160] Oh J.H., Lee S.H. (2011). Prediction of Surface Roughness in Magnetic Abrasive Finishing Using Acoustic Emission and Force Sensor Data Fusion. Proc. Inst. Mech. Eng. Part B J. Eng. Manuf..

[161] Kanish T.C., Kuppan P., Narayanan S., Ashok S.D. (2014). A Fuzzy Logic Based Model to Predict the Improvement in Surface Roughness in Magnetic Field Assisted Abrasive Finishing. Procedia Eng..

[162] Pandiyan V., Tjahjowidodo T., Samy M.P. (2016). In-Process Surface Roughness Estimation Model for Compliant Abrasive Belt Machining Process. Procedia CIRP.

[163] Khalick Mohammad A., Hong J., Wang D. (2017). Polishing of Uneven Surfaces Using Industrial Robots Based on Neural Network and Genetic Algorithm. Int. J. Adv. Manuf. Technol..

[164] Wang R., Cheng M.N., Loh Y.M., Wang C., Fai Cheung C. (2022). Ensemble Learning with a Genetic Algorithm for Surface Roughness Prediction in Multi-Jet Polishing. Expert Syst. Appl..

[165] Fountas N.A., Vaxevanidis N.M. (2021). Optimization of Abrasive Flow Nano-Finishing Processes by Adopting Artificial Viral Intelligence. J. Manuf. Mater. Process..

[166] Caggiano A., Teti R., Alfieri V., Caiazzo F. (2021). Automated Laser Polishing for Surface Finish Enhancement of Additive Manufactured Components for the Automotive Industry. Prod. Eng. Res. Devel..

[167] Speidel A., Sélo R., Bisterov I., Mitchell-Smith J., Clare A.T. (2021). Post Processing of Additively Manufactured Parts Using Electrochemical Jet Machining. Mater. Lett..

[168] Sealy M.P., Madireddy G., Williams R.E., Rao P., Toursangsaraki M. (2018). Hybrid Processes in Additive Manufacturing. J. Manuf. Sci. Eng..

[169] Motallebi R., Savaedi Z., Mirzadeh H. (2022). Post-Processing Heat Treatment of Lightweight Magnesium Alloys Fabricated by Additive Manufacturing: A Review. J. Mater. Res. Technol..

[170] Merklein M., Junker D., Schaub A., Neubauer F. (2016). Hybrid Additive Manufacturing Technologies—An Analysis Regarding Potentials and Applications. Phys. Procedia.

[171] Flynn J.M., Shokrani A., Newman S.T., Dhokia V. (2016). Hybrid Additive and Subtractive Machine Tools—Research and Industrial Developments. Int. J. Mach. Tools Manuf..

[172] LENS 860 Machine Tool Systems—Optomec. https://optomec.com/3d-printed-metals/lens-printers/additive-and-hybrid-manufacturing-860-printer/.

[173] LASERTEC 65 DED Hybrid—ADDITIVE MANUFACTURING Machines by DMG MORI. https://en.dmgmori.com/products/machines/additive-manufacturing/powder-nozzle/lasertec-65-ded-hybrid.

[174] AM—Additive Manufacturing. https://english.mazak.jp/machines/technology/hybrid-multi-tasking-machine/am/#am-l.

[175] K.G.& Co.K (2023). Maschinenfabrik Berthold Hermle AG—Hermle MPA Technology—Additive Manufacturing, Milling at Its Best. https://www.hermle.de/en/services/additive_manufacturing.

[176] Hybrid Additive Manufacturing Archives | Fabrisonic. https://fabrisonic.com/tag/hybrid-additive-manufacturing//.

[177] Hybrid Wire Arc Additive Manufacturing (H-WAAM) | Additive Manufacturing | 3D Metalforge | 3D Metalforge Pte Ltd.—News. https://3dmetalforge.com/news/?limit=20&limitstart=20.

[178] Products | Hybrid Manufacturing Technologies. https://hybridmanutech.com/products/.

[179] 3D HYBRID: AM FOR CNC—Home. https://www.3dhybridsolutions.com/.

[180] Ren L., Padathu A.P., Ruan J., Sparks T., Liou F. Three Dimensional Die Repair Using a Hybrid Manufacturing System. Proceedings of the 17th Annual Solid Freeform Fabrication Symposium.

[181] Jones J., McNutt P., Tosi R., Perry C., Wimpenny D. (2012). Remanufacture of Turbine Blades by Laser Cladding, Machining and In-Process Scanning in a Single Machine.

[182] Stavropoulos P., Bikas H., Avram O., Valente A., Chryssolouris G. (2020). Hybrid Subtractive–Additive Manufacturing Processes for High Value-Added Metal Components. Int. J. Adv. Manuf. Technol..

[183] Le V.T., Paris H., Mandil G. (2018). The Development of a Strategy for Direct Part Reuse Using Additive and Subtractive Manufacturing Technologies. Addit. Manuf..

[184] Feldhausen T., Heinrich L., Saleeby K., Burl A., Post B., MacDonald E., Saldana C., Love L. (2022). Review of Computer-Aided Manufacturing (CAM) Strategies for Hybrid Directed Energy Deposition. Addit. Manuf..

[185] Feldhausen T., Saleeby K., Kurfess T. (2021). Spinning the Digital Thread with Hybrid Manufacturing. Manuf. Lett..

[186] Lynn R., Louhichi W., Parto M., Wescoat E., Kurfess T. (2017). Rapidly Deployable MTConnect-Based Machine Tool Monitoring Systems. Proceedings of the Volume 3: Manufacturing Equipment and Systems.

[187] Akin S., Lee S., Jo S., Ruzgar D.G., Subramaniam K., Tsai J.-T., Jun M.B.-G. (2022). Cold Spray-Based Rapid and Scalable Production of Printed Flexible Electronics. Addit. Manuf..

[188] Prashar G., Vasudev H. (2021). A Comprehensive Review on Sustainable Cold Spray Additive Manufacturing: State of the Art, Challenges and Future Challenges. J. Clean Prod..

[189] Yu T., Chen M., Wu Z. (2022). Experimental and Numerical Study of Deposition Mechanisms for Cold Spray Additive Manufacturing Process. Chin. J. Aeronaut..

[190] Cold Spray Additive Manufacturing | Call Us Today 514 865-5763. https://polycontrols.com/polycsam/.

[191] Stavropoulos P., Foteinopoulos P., Stavridis J., Bikas H. (2023). Increasing the Industrial Uptake of Additive Manufacturing Processes: A Training Framework. Adv. Ind. Manuf. Eng..

[192] Ahuett-Garza H., Kurfess T. (2018). A Brief Discussion on the Trends of Habilitating Technologies for Industry 4.0 and Smart Manufacturing. Manuf. Lett..

[193] Blakey-Milner B., Gradl P., Snedden G., Brooks M., Pitot J., Lopez E., Leary M., Berto F., du Plessis A. (2021). Metal Additive Manufacturing in Aerospace: A Review. Mater. Des..

[194] Debnath B., Shakur M.S., Tanjum F., Rahman M.A., Adnan Z.H. (2022). Impact of Additive Manufacturing on the Supply Chain of Aerospace Spare Parts Industry—A Review. Logistics.

[195] Schweiger J., Bomze D., Schwentenwein M. (2019). 3D Printing of Zirconia–What Is the Future?. Curr. Oral Health Rep..

[196] Eyers D., Dotchev K. (2010). Technology Review for Mass Customisation Using Rapid Manufacturing. Assem. Autom..

[197] Gardner L., Kyvelou P., Herbert G., Buchanan C. (2020). Testing and Initial Verification of the World’s First Metal 3D Printed Bridge. J. Constr. Steel Res..

[198] Selema A., Ibrahim M.N., Sergeant P. (2022). Electrical Machines Winding Technology: Latest Advancements for Transportation Electrification. Machines.

[199] Jankovics D., Barari A. (2019). Customization of Automotive Structural Components Using Additive Manufacturing and Topology Optimization. IFAC-PapersOnLine.

[200] Jeng J.Y., Lin M.C. (2001). Mold Fabrication and Modification Using Hybrid Processes of Selective Laser Cladding and Milling. J. Mater. Process. Technol..

[201] Binder M., Illgner M., Anstaett C., Kindermann P., Kirchbichler L., Seidel C. (2018). Automated Manufacturing of Sensor-Monitored Parts. Laser Tech. J..

[202] Korkmaz M.E., Waqar S., Garcia-Collado A., Gupta M.K., Krolczyk G.M. (2022). A Technical Overview of Metallic Parts in Hybrid Additive Manufacturing Industry. J. Mater. Res. Technol..

[203] Yasir M., Danish M., Mia M., Gupta M.K., Sarikaya M. (2021). Investigation into the Surface Quality and Stress Corrosion Cracking Resistance of AISI 316L Stainless Steel via Precision End-Milling Operation. Int. J. Adv. Manuf. Technol..

[204] Prathipati R., Dora S.P., Chanamala R. (2020). Wear Behavior of Wire Electric Discharge Machined Surface of 316L Stainless Steel. SN Appl. Sci..

[205] Larimian T., AlMangour B., Grzesiak D., Walunj G., Borkar T. (2021). Effect of Laser Spot Size, Scanning Strategy, Scanning Speed, and Laser Power on Microstructure and Mechanical Behavior of 316L Stainless Steel Fabricated via Selective Laser Melting. J. Mater. Eng. Perform..

[206] Marya M., Singh V., Marya S., Hascoet J.Y. (2015). Microstructural Development and Technical Challenges in Laser Additive Manufacturing: Case Study with a 316L Industrial Part. Metall. Mater. Trans. B Process Metall. Mater. Process. Sci..

[207] Wang L., Xue J., Wang Q. (2019). Correlation between Arc Mode, Microstructure, and Mechanical Properties during Wire Arc Additive Manufacturing of 316L Stainless Steel. Mater. Sci. Eng. A.

[208] Rännar L.E., Koptyug A., Olsén J., Saeidi K., Shen Z. (2017). Hierarchical Structures of Stainless Steel 316L Manufactured by Electron Beam Melting. Addit. Manuf..

[209] Pacheco J.T., Meura V.H., Bloemer P.R.A., Veiga M.T., de Moura Filho O.C., Cunha A., Teixeira M.F. (2022). Laser Directed Energy Deposition of AISI 316L Stainless Steel: The Effect of Build Direction on Mechanical Properties in as-Built and Heat-Treated Conditions. Adv. Ind. Manuf. Eng..

[210] Sadaf M., Bragaglia M., Nanni F. (2021). A Simple Route for Additive Manufacturing of 316L Stainless Steel via Fused Filament Fabrication. J. Manuf. Process..

[211] Yang Y., Gong Y., Qu S., Xin B., Xu Y., Qi Y. (2019). Additive/Subtractive Hybrid Manufacturing of 316L Stainless Steel Powder: Densification, Microhardness and Residual Stress. J. Mech. Sci. Technol..

[212] Kaynak Y., Kitay O. (2018). Porosity, Surface Quality, Microhardness and Microstructure of Selective Laser Melted 316L Stainless Steel Resulting from Finish Machining. J. Manuf. Mater. Process..

[213] Lynn R., Helu M., Sati M., Tucker T., Kurfess T. (2020). The State of Integrated Computer-Aided Manufacturing/Computer Numerical Control: Prior Development and the Path Toward a Smarter Computer Numerical Controller. Smart Sustain. Manuf. Syst..

[214] Xu X.W., Newman S.T. (2006). Making CNC Machine Tools More Open, Interoperable and Intelligent—A Review of the Technologies. Comput. Ind..

[215] Latif K., Adam A., Yusof Y., Kadir A.Z.A. (2021). A Review of G Code, STEP, STEP-NC, and Open Architecture Control Technologies Based Embedded CNC Systems. Int. J. Adv. Manuf. Technol..

[216] Wang R., Gu C., He S., Shi Z., Meng W. (2022). An Interoperable and Flat Industrial Internet of Things Architecture for Low Latency Data Collection in Manufacturing Systems. J. Syst. Archit..

[217] Möhring H.-C., Wiederkehr P., Erkorkmaz K., Kakinuma Y. (2020). Self-Optimizing Machining Systems. CIRP Ann..

[218] Everton S.K., Hirsch M., Stravroulakis P., Leach R.K., Clare A.T. (2016). Review of In-Situ Process Monitoring and in-Situ Metrology for Metal Additive Manufacturing. Mater. Des..

[219] Usha S. (2021). In Situ Monitoring of Metal Additive Manufacturing Process: A Review. Additive Manufacturing.

[220] Goh G.D., Sing S.L., Yeong W.Y. (2021). A Review on Machine Learning in 3D Printing: Applications, Potential, and Challenges. Artif. Intell. Rev..

[221] AbouelNour Y., Gupta N. (2022). In-Situ Monitoring of Sub-Surface and Internal Defects in Additive Manufacturing: A Review. Mater. Des..

[222] De Baere D., Strantza M., Hinderdael M., Devesse W., Guillaume P. Effective Structural Health Monitoring with Additive Manufacturing. Proceedings of the EWSHM-7th European Workshop on Structural Health Monitoring.

[223] Park S.-H., Choi S., Song D.-G., Jhang K.-Y. (2022). Microstructural Characterization of Additively Manufactured Metal Components Using Linear and Nonlinear Ultrasonic Techniques. Materials.

[224] Qi X., Chen G., Li Y., Cheng X., Li C. (2019). Applying Neural- Network- Based Machine Learning to Additive Manufacturing: Current Applications, Challenges, and Future Perspectives. Engineering.

[225] Jithinraj E.K., Ahmed A., Boban J. (2022). Improving the Surface Integrity of Additively Manufactured Curved and Inclined Metallic Surfaces Using Thermo-Electric Energy Assisted Polishing. Surf. Coat Technol..

[226] Xu P., Cheung C.F., Wang C., Zhao C. (2020). Novel Hybrid Robot and Its Processes for Precision Polishing of Freeform Surfaces. Precis. Eng..

[227] Bonnard R., Hascoët J.-Y., Mognol P., Stroud I. (2018). STEP-NC Digital Thread for Additive Manufacturing: Data Model, Implementation and Validation. Int. J. Comput. Integr. Manuf..

[228] Pasi B.N., Mahajan S.K., Rane S.B. (2022). Development of Innovation Ecosystem Framework for Successful Adoption of Industry 4.0 Enabling Technologies in Indian Manufacturing Industries. J. Sci. Technol. Policy Manag..

[229] Tuloup C., Harizi W., Aboura Z., Meyer Y., Khellil K., Lachat R. (2019). On the Use of In-Situ Piezoelectric Sensors for the Manufacturing and Structural Health Monitoring of Polymer-Matrix Composites: A Literature Review. Compos. Struct..

[230] Nasir V., Sassani F. (2021). A Review on Deep Learning in Machining and Tool Monitoring: Methods, Opportunities, and Challenges. Int. J. Adv. Manuf. Technol..

[231] Liu J., Zhou H., Liu X., Tian G., Wu M., Cao L., Wang W. (2019). Dynamic Evaluation Method of Machining Process Planning Based on Digital Twin. IEEE Access.

[232] Schoop J., Poonawala H.A., Adeniji D., Clark B. (2021). AI-Enabled Dynamic Finish Machining Optimization for Sustained Surface Integrity. Manuf. Lett..

[233] Shen B., Erol O., Fang L., Kang S.H. (2020). Programming the Time into 3D Printing: Current Advances and Future Directions in 4D Printing. Multifunct. Mater..

[234] Shin D.G., Kim T.H., Kim D.E. (2017). Review of 4D Printing Materials and Their Properties. Int. J. Precis. Eng. Manuf. -Green Technol. 2017 4:3.

[235] Ge Q., Sakhaei A.H., Lee H., Dunn C.K., Fang N.X., Dunn M.L. (2016). Multimaterial 4D Printing with Tailorable Shape Memory Polymers. Sci. Rep..

[236] Momeni F., M. Mehdi Hassani N.S., Liu X., Ni J. (2017). A Review of 4D Printing. Mater. Des..

[237] Pugliese R., Regondi S. (2022). Artificial Intelligence-Empowered 3D and 4D Printing Technologies toward Smarter Biomedical Materials and Approaches. Polymers.

[238] Pugliese R., Regondi S., Marini R. (2021). Machine Learning-Based Approach: Global Trends, Research Directions, and Regulatory Standpoints. Data Sci. Manag..

[239] Huff T.J., Ludwig P.E., Zuniga J.M. (2018). The Potential for Machine Learning Algorithms to Improve and Reduce the Cost of 3-Dimensional Printing for Surgical Planning. Expert Rev. Med. Devices.

[240] Quanjin M., Rejab M.R.M., Idris M.S., Kumar N.M., Abdullah M.H., Reddy G.R. (2020). Recent 3D and 4D Intelligent Printing Technologies: A Comparative Review and Future Perspective. Procedia Comput. Sci..

[241] Kusiak A. (2019). Fundamentals of Smart Manufacturing: A Multi-Thread Perspective. Annu. Rev. Control.

[242] Rahman M.A., Shakur M.S., Ahamed S., Hasan S., Rashid A.A., Islam M.A., Haque S.S., Ahmed A. (2022). A Cloud-Based Cyber-Physical System with Industry 4.0: Remote and Digitized Additive Manufacturing. Automation.

[243] Tripathi S., Gupta M. (2020). A Framework for Procurement Process Re-Engineering in Industry 4.0. BPMJ.

[244] Lee S.M., Lee D., Kim Y.S. (2019). The Quality Management Ecosystem for Predictive Maintenance in the Industry 4.0 Era. Int. J. Qual. Innov..

[245] Afanasev M.Y., Fedosov Y.V., Krylova A.A., Shorokhov S.A. An Application of Blockchain and Smart Contracts for Machine-to-Machine Communications in Cyber-Physical Production Systems. Proceedings of the 2018 IEEE Industrial Cyber-Physical Systems (ICPS).

[246] Deebak B.D., AL-Turjman F. (2022). A Robust and Distributed Architecture for 5G-Enabled Networks in the Smart Blockchain Era. Comput. Commun..

[247] Borangiu T., Trentesaux D., Thomas A., Leitão P., Barata J. (2019). Digital Transformation of Manufacturing through Cloud Services and Resource Virtualization. Comput. Ind..

[248] Wang L., Wang X.V., Gao L., Váncza J. (2014). A Cloud-Based Approach for WEEE Remanufacturing. CIRP Ann..

[249] Adamson G., Wang L., Holm M., Moore P. (2015). Cloud Manufacturing—A Critical Review of Recent Development and Future Trends. Int. J. Comput. Integr. Manuf..

[250] Altintas Y., Brecher C., Weck M., Witt S. (2005). Virtual Machine Tool. CIRP Ann..

[251] Cai Y., Starly B., Cohen P., Lee Y.-S. (2017). Sensor Data and Information Fusion to Construct Digital-Twins Virtual Machine Tools for Cyber-Physical Manufacturing. Procedia Manuf..

[252] Xu X. (2017). Machine Tool 4.0 for the New Era of Manufacturing. Int. J. Adv. Manuf. Technol..

[253] Stavropoulos P. (2022). Digitization of Manufacturing Processes: From Sensing to Twining. Technologies.

[254] Zhang J., Deng C., Zheng P., Xu X., Ma Z. (2021). Development of an Edge Computing-Based Cyber-Physical Machine Tool. Robot. Comput. -Integr. Manuf..

[255] Liu C., Zheng P., Xu X. (2021). Digitalisation and Servitisation of Machine Tools in the Era of Industry 4.0: A Review. Int. J. Prod. Res..

[256] Shakur M.S., Islam M.A., Rahman M.A. A Cyber Physical Industry 4.0 Framework of Image Based Defect Detection for Additive Manufacturing. Proceedings of the 2021 International Conference on Computer, Communication, Chemical, Materials and Electronic Engineering (IC4ME2).

[257] Gunasegaram D.R., Murphy A.B., Barnard A., DebRoy T., Matthews M.J., Ladani L., Gu D. (2021). Towards Developing Multiscale-Multiphysics Models and Their Surrogates for Digital Twins of Metal Additive Manufacturing. Addit. Manuf..

[258] Yavari R., Riensche A., Tekerek E., Jacquemetton L., Halliday H., Vandever M., Tenequer A., Perumal V., Kontsos A., Smoqi Z. (2021). Digitally Twinned Additive Manufacturing: Detecting Flaws in Laser Powder Bed Fusion by Combining Thermal Simulations with in-Situ Meltpool Sensor Data. Mater. Des..

[259] Gunasegaram D.R., Murphy A.B., Matthews M.J., DebRoy T. (2021). The Case for Digital Twins in Metal Additive Manufacturing. J. Phys. Mater..

[260] Liu C., le Roux L., Körner C., Tabaste O., Lacan F., Bigot S. (2022). Digital Twin-Enabled Collaborative Data Management for Metal Additive Manufacturing Systems. J. Manuf. Syst..

[261] Gupta P., Krishna C., Rajesh R., Ananthakrishnan A., Vishnuvardhan A., Patel S.S., Kapruan C., Brahmbhatt S., Kataray T., Narayanan D. (2022). Industrial Internet of Things in Intelligent Manufacturing: A Review, Approaches, Opportunities, Open Challenges, and Future Directions. Int. J. Interact. Des. Manuf..

[262] Kim J., Takahashi H., Miyashita H., Annett M., Yeh T. (2017). Machines as Co-Designers: A Fiction on the Future of Human-Fabrication Machine Interaction. Proceedings of the 2017 CHI Conference Extended Abstracts on Human Factors in Computing Systems.

[263] Ardanza A., Moreno A., Segura Á., de la Cruz M., Aguinaga D. (2019). Sustainable and Flexible Industrial Human Machine Interfaces to Support Adaptable Applications in the Industry 4.0 Paradigm. Int. J. Prod. Res..

[264] Reisch R.T., Hauser T., Lutz B., Tsakpinis A., Winter D., Kamps T., Knoll A. (2022). Context Awareness in Process Monitoring of Additive Manufacturing Using a Digital Twin. Int. J. Adv. Manuf. Technol..

[265] Morgan J., Halton M., Qiao Y., Breslin J.G. (2021). Industry 4.0 Smart Reconfigurable Manufacturing Machines. J. Manuf. Syst..

[266] Borish M., Westfall J. Additive and Subtractive Manufacturing Augmented Reality Interface (ASMARI). Proceedings of the 2020 SoutheastCon.

[267] Phua A., Davies C.H.J., Delaney G.W. (2022). A Digital Twin Hierarchy for Metal Additive Manufacturing. Comput. Ind..

[268] Phua A., Delaney G.W., Cook P.S., Davies C.H.J. Intelligent Digital Twins Can Accelerate Scientific Discovery and Control Complex Multi-Physics Processes. Proceedings of the ICML 2022 2nd AI for Science Workshop.

